# Bioactive Polymeric Composites for Tooth Mineral Regeneration: Physicochemical and Cellular Aspects

**DOI:** 10.3390/jfb2030271

**Published:** 2011-09-14

**Authors:** Drago Skrtic, Joseph M. Antonucci

**Affiliations:** 1 Paffenbarger Research Center, American Dental Association Foundation; Gaithersburg, MD 20899, USA; 2 Polymers Division, National Institute of Standards and Technology; Gaithersburg, MD 20899, USA; E-Mail: joseph.antonucci@nist.gov

**Keywords:** amorphous calcium phosphate, bioactive dental composites, cellular responses, mineral regeneration, physicochemical characterization

## Abstract

Our studies of amorphous calcium phosphate (ACP)-based dental materials are focused on the design of bioactive, non-degradable, biocompatible, polymeric composites derived from acrylic monomer systems and ACP by photochemical or chemically activated polymerization. Their intended uses include remineralizing bases/liners, orthodontic adhesives and/or endodontic sealers. The bioactivity of these materials originates from the propensity of ACP, once exposed to oral fluids, to release Ca and PO_4_ ions (building blocks of tooth and bone mineral) in a sustained manner while spontaneously converting to thermodynamically stable apatite. As a result of ACP's bioactivity, local Ca- and PO_4_-enriched environments are created with supersaturation conditions favorable for the regeneration of tooth mineral lost to decay or wear. Besides its applicative purpose, our research also seeks to expand the fundamental knowledge base of structure-composition-property relationships existing in these complex systems and identify the mechanisms that govern filler/polymer and composite/tooth interfacial phenomena. In addition to an extensive physicochemical evaluation, we also assess the leachability of the unreacted monomers and *in vitro* cellular responses to these types of dental materials. The systematic physicochemical and cellular assessments presented in this study typically provide model materials suitable for further animal and/or clinical testing. In addition to their potential dental clinical value, these studies suggest the future development of calcium phosphate-based biomaterials based on composite materials derived from biodegradable polymers and ACP, and designed primarily for general bone tissue regeneration.

## 1. Introduction

### 1.1. Hard Tissue Regenerating Materials Based on Calcium Phosphates

With the exception of a small portion of the inner ear, all hard tissues of the human body are formed of calcium phosphate(s) CaPs [1]. CaPs also occur in pathological calcifications (dental and urinary calculi, atherosclerotic lesions). The atomic arrangements of CaPs are built up around an orthophosphate (PO_4_) network that gives stability to their structures. Due to the tripotic equilibrium existing in their aqueous solutions, variations in pH strongly affect the relative concentrations of the orthophosphoric acid polymorphs (H_3_PO_4_, H_2_PO_4_^−^, HPO_4_^2−^ and PO_4_^3−^) and, consequently, the chemical composition of CaP that forms by spontaneous precipitation from supersaturated solutions. The general rule in the CaP family is: the lower the Ca/PO_4_ molar ratio, the more acidic and water-soluble the CaP. Structural and chemical properties of CaPs have been extensively reviewed [1,2,3,4]. Eleven crystalline and one amorphous form of CaPs with different Ca/P molar ratios, solubility and crystallographic properties have been identified, seven of which are biologically-relevant (Table 1). The *in vivo* presence of small peptides, proteins and inorganic additives considerably influences CaP formation, making it difficult to predict the possible phases that may form. The least soluble, HAP, preferentially formed under neutral and basic conditions, is usually non-stoichiometric. DCPD and OCP are detected primarily under pathological conditions. In normal *in vivo* calcifications, the formation of ACP followed by conversion to HAP has been suggested [1,2,3,4].

A variety of CaP-based, hard tissue restorative materials have been proposed based primarily on the compositional similarity of CaPs to the inorganic constituents of bone and teeth. Because of their inherent rigidity and brittleness, CaP biomaterials are primarily designed for filling bone defects in oral and orthopedic surgery or coatings for dental implants and metallic prostheses [1,2]. Enamel, considered to be the most fracture-resistant and tough inorganic material in the biological world, contains no cells in an adult body, and once degraded cannot be regenerated via biological processes. All biological hard tissues appear to be very complex composites of an inorganic mineral phase (provides strength) and organic phase (contributes to toughness). There is an abundance of information on a large variety of bone substituting composites made of CaPs (ACP, OCP, TCPs, HAP, rarely FAP) and organic polymers (both biostable and biodegradable [1]). Organic polymers are either synthetic (e.g., polyesters, polyepoxides, polyacrylics, poly-ε-caprolactones, *etc.*) or of biological origin (e.g., collagen, gelatin, chitosan, alginate, modified starch, cellulose esters).

**Table 1:** Chemical and thermodynamic properties of biologically relevant calcium phosphate(s) (CaPs) [1,2,3,3].

**Name (acronym)**	**Formula**	**Ca/P**	**pK_sp_#**	**pH stability range##**
Dicalcium phosphate dihydrate (DCPD)	CaHPO_4_·2H_2_O	1.00	6.6	2.0–6.0
Octacalcium phosphate (OCP)	Ca_8_(HPO_4_)_2_(PO_4_)_4_·5H_2_O	1.33	96.6	5.5–7.0
Amorphous calcium phosphate (ACP)	Ca_3_(PO_4_)_2_·nH_2_O*	1.50 @	Nd	∼5.0–12.0**
α-tricalcium phosphate (α-TCP)	Ca_3_(PO_4_)_2_	1.50	25.5	N/a
β-tricalcium phosphate (β-TCP)	Ca_3_(PO_4_)_2_	1.50	28.9	N/a
Hydroxyapatite (HAP)	Ca_10_(PO_4_)_6_(OH)_2_	1.67	116.8	9.5–12.0
Fluoroapatite (FAP)	Ca_10_(PO_4_)_6_F_2_	1.67	120.0	7.0–12.0

* Approximate formula; n = 3.0–4.5.

# pK_sp_ is the negative logarithm of the ionic product for the given formula at 25 °C (hydrate water excluded).

## In agueous solutions at 25 °C.

** Always metastable.

@ Average value for ACP synthesized in our group; reported values vary from 1.2 to 2.2 (1, 3). Nd—could not be determined precisely; reported values range from 25.7 to 32.7. N/a—not applicable; α-TCP and β-TCP cannot be precipitated from aqueous solutions. Dissolution rate in acidic buffer decreases as follows: ACP ≫ α-TCP ≫ β-TCP > non-stoichiometric HAP > HAP > FAP.

*In vivo*, the interactions between the biomaterial and its “bioenvironment” are multifaceted due to non-equilibrium conditions and the undefined nature and amounts of compounds participating in these interactions. It has recently been documented that controlled release of the ionic dissolution products of bioactive materials results in regeneration of tissues [5]. Such controlled release of soluble moieties from bioactive filler/resorbable polymer composites leading to gene activation provides the conceptual basis for the molecular design of biomaterials optimized for in situ tissue regeneration. However, the exact mechanism of CaPs bioactivity is not yet well-understood. The majority of bioresearchers have embraced a concept introduced by Prof. Hench [6] based on bio-glasses which entails 11 successive reactions steps. The initial five steps are chemical in nature (hydrolysis, formation of Si-OH bonds, poly-condensation reactions, formation of amorphous CaP compound, and crystallization to HAP) resulting in the formation of bone-like apatite. In the case of experimental ACP-based materials, formation of bone-like apatite would be spurred by the spontaneous, *in situ* conversion of ACP, instead of the formation of an amorphous precursor via negatively charged hydroxyl, carbonyl and/or phosphate functionalities as is the case with bio-glasses and other types of synthetic CaP biomaterials [1,2]. The six biological steps (adsorption of biological moieties in HAP layer, action of macrophages, cell attachment and differentiation, generation and maturation of matrix) ultimately lead to the formation of new bone.

The cells that are most directly related to bone formation and regeneration are [7]: osteoblasts, osteoclasts, mesenchymal stem cells and osteoprogenitor cells. Osteoblasts differ substantially in their properties depending upon their stage of development. Active osteoblasts (cuboidal in shape, mononuclear, rich in alkaline phosphatase (ALP) activity) synthesize and secrete collagen type I, glycoproteins (osteocalcin and osteopontin), cytokines and growth factors into a region of unmineralized matrix between the cell body and the mineralized matrix [8], and produce CaP minerals both intra- and extra-cellularly within vesicles [9]. *In vitro* cellular-biomaterial interactions are assayed by osteoblasts, osteocarcoma cell lines or mesenchymal-osteoprogenitor cells [7]. The osteoprogenitor cells are suggested because they participate at an early stage of new bone formation *in vivo* [10]. Osteoblasts are in intimate contact with CaP surfaces via production of extracellular collagen, which is firmly attached to the substrate [9]. Their active participation in biomineralization [9,11] cannot, however, be assayed on CaP biomaterials because of the similarities in composition between the substrate and the extracellular matrix [7].

CaP-related parameters that can affect cellular activity are: dissolution-precipitation behavior, chemical composition, topography and surface energy [12,13,14,15,16,17,18,19,20,21]. In particular, a clear link has been demonstrated between the levels of free Ca and PO_4_ ions in culture medium and osteoblastic activities [12,13,14,15,16], as well as early bone formation *in vitro* and *in vivo* [7,8]. Any change in Ca/P ratio as a consequence of compositional changes in the CaP phase directly affects ion exchange mechanisms [10]. On the other hand, the presence of carbonate in the apatitic network of the biomaterial had deleterious effects on osteoblast proliferation and ALP production [13,19]. Grooved CaP surfaces influence the osteoblastic guidance regardless of the nature of substrate [11]. In contact with both micro- and macro-porous CaP ceramic, osteoblasts can bridge pores many times larger than their full length [9]. Osteoblasts are also sensitive to the shape and size of apatite crystals [21]. Their initial proliferation activity can be affected by the surface energy, although the energy factor appears to be of lesser significance at the latter stages of osteoblastic activity [7,19]. Essentially the same physicochemical processes, *i.e.*, dissolution kinetics, compositional effects (presence of mineral ions and/or carbonate), surface energy and surface roughness govern CaP ceramics/osteoclasts interactions and, ultimately, also control the loss of bone mineral [19,22,23,24,25,26,27]. In this article, only studies related to the osteoblast/CaP interactions are considered; the activities involved in wound blood clots have not been discussed.

### 1.2. ACP-based Dental Materials

Among CaP biomaterials, ACP-based ones are particularly attractive due to ACP's metastability in a wide range of pHs in aqueous solutions ([1,2]; Table 1), which provides the extended supply of Ca and PO_4_ ions needed for the repair of tooth mineral structures damaged by dental caries and wear. Traditionally, caries prevention strategies are focused on reducing bacterial growth, neutralizing oral acids and using various remineralizing agents, such as remineralizing ions-delivering dentifrices, chewing gums and mouthwashes, and systemic and/or topical fluoridation. Mineral restoration by remineralizing solutions containing calcium and phosphate ions often fails clinically because of the low solubility of calcium phosphates, particularly in the presence of fluoride ions, and inability of calcium and phosphate ions to incorporate into plaque and localize at the tooth surface [28]. Incorporation of fluoride into tooth mineral as FAP or fluoride-enriched HAP decreases the solubility of tooth enamel and is viewed as the cornerstone strategy for caries prevention [29,30]. However, fluoride is less effective as a dentin remineralizing vehicle [31,32]. Recently, in addition to the various fluoride treatments, two distinct new remineralization technologies have been introduced: (a) casein-phosphopeptide stabilized ACP in the form of mouthrinses and sugar-free chewing gums [28,33,34]; and (b) ACP-based polymeric composites [35,36,37]. Other dental applications of ACP include varnishes, dentifrices and desensitizing agents [38].

The physicochemical properties of ACP-based dental composites, typically formulated from various methacrylic resin matrices and ACP filler are a result of inter-atomic or molecular interactions between the organic matrix and the ACP filler particles. In order to perceive the potential for interactions of individual components of these materials and the oral environment, it is essential to understand the chemistry of composites. It is also important to consider the biodegradation of composite/adhesive chemistries at the interface with tooth structures. The susceptibility to degradation (inherent in the choice of chemistries selected for the formulation of dental composites) is promoted by salivary enzymes and related co-factors, generating defined chemical products [39]. The latter products may modulate the biological activity of cells and oral bacteria which interface with restorative material.

Polymerization of dental resin composites is usually less complete than that of the unfilled resin, and almost every organic component can be detected in the extracts of polymerized materials [40,41]. The chemical structure/property relations of the constituent monomers, compositional differences involving polymers and initiator systems, and the attainable degree of vinyl conversion (DVC), especially as it relates to the leachable monomers, are important contributing factors that control the cellular response. Cytotoxicity is most likely to depend on leachable residual monomers and other leachable organic species from the cured composite. Therefore, to better understand the correlation between the cytotoxicity and the DVC, it seems prudent to perform the analysis of leachable organic moieties as an integral part of composite's evaluation.

The underlying working hypotheses of our investigations are: (1) that the physicochemical properties of ACP composites can be tailored through the choice of resin system and by improved ACP's dispersion throughout the composite, and (2) that biocompatibility concerns could be minimized by improving the DVC attained in the composites. In this paper, we describe the comprehensive physicochemical evaluation of ACP composites formulated for various dental applications and cellular responses to these materials. The lessons learned from the structure-composition-property relationships existing in these complex systems for dental applications may be of seminal value in the foreseen development of ACP-based materials intended for general bone recovery.

## 2. Experimental Section

Methods and techniques used in physicochemical and cellular assessment of ACP composites are compiled in Table 2. The fundamentals of the experimental protocols are provided in the corresponding subsections.

**Table 2:** Methods and techniques employed in physicochemical and biological characterization of amorphous calcium phosphate (ACP) fillers, monomers, unfilled resins (copolymers) and ACP composites. Indicated acronyms will be used throughout this article.

	**Physicochemical evaluation**
Atomic emission spectroscopy (AES)	Compositional analysis of ACP fillers (Ca/PO_4_ ratio of the solid); kinetics of Ca and PO_4_ ions release from composites
Dilatometry	Volumetric changes of composite specimens as a consequence of polymerization shrinkage (PS)
Fourier-transform infrared (FTIR) spectroscopy and microspectroscopy (FTIR-m)	Validation of ACP structure; distribution of the resin and ACP filler on composite's surface; degree of vinyl conversion (DVC) of copolymers and composites
Gravimetry	The overall mass changes resulting from water sorption, filler dissolution and leachability of the unreacted species; water uptake and hygroscopic expansion (HE) of copolymers and composites upon exposure to relative humidity (RH) or aqueous immersion
Mechanical tests	Biaxial flexure strength (BFS), shear bond strength (SBS) of copolymer and composite specimens in dry and wet state
Nuclear magnetic resonance (^1^H NMR) spectroscopy	Identification and quantification of leachables in the extracts of copolymers and composites
Particle size distribution (PSD) analysis	Histograms of the volume and number PSD; size range and median diameter (d_m_) of ACP filler
Scanning electron microscopy (SEM)	Morphology and topology of ACP filler, surface characteristics of copolymers and composites before and after aqueous immersion
Tensometry	Determination of the stresses developing within the composites due to shrinkage upon polymerization (PSS)
Thermogravimetric analysis (TGA)	Temperature-dependent mass changes of the fillers and composites
X-ray diffraction (XRD)	Long-range (non)crystalline order of the fillers and their stability upon aqueous immersion; intra-composite ACP to HAP transformation
	**Cellular responses**
Colorimetry	Viability of cells exposed to the extracts from copolymer and/or composite specimens
Phase contrast microscopy	Effects of the copolymer and composite extracts on cell morphology

Note: Due to a large number of acronyms used throughout this manuscript, List of Acronyms is provided in the Appendix for the reader's convenience.

### 2.1. Synthesis, Modification and Characterization of ACP Fillers

ACP precipitated instantaneously in a closed system at 23 °C upon rapidly mixing equal volumes of a 80 mmol/L Ca(NO_3_)_2_ solution and a 54 mmol/L Na_2_HPO_4_ solution that contained a molar fraction of 2% Na_4_P_2_O_7_, a known inhibitor of HAP formation. Various additives were introduced during ACP synthesis {cations (at a mole fraction of 10% based on the Ca reactant [42]), surfactants (at 0.05% or 0.10% by mass) or polymers (at a 0.25% by mass [43])} as surface modifiers that were expected to reduce agglomeration of ACP and potentially render fillers with narrower, more homogeneous particle size distribution (PSD) and enhanced hydrolytic stability. The reaction pHs of the precipitating systems were maintained between 8.5 and 9.0. The suspensions were filtered, the solid phases washed subsequently with ice-cold ammoniated water and acetone, freeze-dried and then lyophilized. Dry solids were then used as-synthesized ACP (as-made or am-ACP) or were further treated as follows. In addition to the surface-modification by various additives, silanization of ACP with 3-aminopropyltrimethoxysilane (APTMS) or methacryloxypropyltrimethoxysilane (MPTMS) (at a mass fraction of 2% relative to ACP [44]), grinding [45] and mechanical ball milling (planetary ball mill PM 100, Retch Inc., Newton, PA, USA; [46]) were evaluated as alternative ways to break up large ACP agglomerates that regularly form during the spontaneous precipitation of ACP. Detailed descriptions of the experimental protocols employed in ACP surface modification and/or grinding and milling are provided in [42,43,44,45,46]. The main objectives of these studies were to validate the amorphous character of the solids, determine their particle size distribution (PSD) and evaluate their stability upon aqueous exposure.

The ACPs obtained following the grinding and milling protocols were assigned g-ACP and m-ACP, respectively. The amorphous state of all ACPs was verified by powder X-ray diffraction (XRD; DMAX 2000 diffractometer, Rigaku/USA Inc., Danvers, MA, USA) and Fourier-transform spectroscopy (FTIR: Nicolet Magna-IR FTIR 550 spectrophotometer, Nicolet Instrumentation Inc., Madison, WI, USA). XRD patterns were recorded from 4° to 60° 2θ with CuKα radiation (λ = 0.154 nm) at 40 kV and 40 mA. The samples were step-scanned in intervals of 0.010° 2θ at a scanning speed of 1.000 deg/min. The FTIR spectra (4000 cm^−1^ to 400 cm^−1^) were recorded using a KBr pellet technique (0.8 mg to 1.0 mg solid/400 mg KBr). The particle size distribution (PSD) of the ACP fillers was determined by laser light scattering (CIS-100 particle size analyzer, Ankersmid, Metropolitan Computing Corporation, E. Hanover, NJ, USA) in dry or wet state (dispersed in isopropanol and utrasonicated for 10 min at room temperature prior to the analysis). The median particle size diameter (d_m_) values were taken as a primary indicator of alterations in the agglomeration of the ACP particulates (the higher the d_m_ value, the more agglomerated the ACP). Water content and the relative ratio of surface-bound *vs.* structurally incorporated water of ACP fillers were determined by TGA (7 Series Thermal Analysis System, Perkin Elmer, Norwalk, CT, USA) by heating powdered ACP samples (initial weight (5 to 10) mg) at the rate of 20 °/min (temperature range: (30 to 600) °C) in air. The morphology/topology of ACP powders, after the specimens were sputter-coated with gold, was determined by scanning electron microscopy ((SEM), JEOL 35C instrument, JEOL, Inc., Peabody, MA, USA). ACP fillers were stored under vacuum over a dessicant to avoid exposure to humidity and a premature conversion to HAP before being used for fabrication of composites and their physicochemical and biological assessments.

### 2.2. Formulation and Characterization of Experimental Resins

The commercially available base monomers, diluent monomers, adhesive monomers and the polymerization initiator systems used to fabricate experimental resins are listed in Table 3.

**Table 3:** Monomers and the polymerization-initiating systems utilized to formulate the experimental methacrylate resins employed in the studies. Indicated acronyms/commercial names will be used throughout this manuscript (they are also defined in the Appendix).

**Component**	**Acronym/Comm. name**

**Base monomers**	
2,2-Bis(p-2′-hydroxy-3′-methcryloxypropoxy)phenyl-propane	Bis-GMA
Ethoxylated bisphenol A dimethacrylate	EBPADMA
Urethane dimethacrylate	UDMA

**Diluent monomers**	
2-hydroxyethyl methacrylate	HEMA
Hexamethylene dimethacrylate	HmDMA
Poly(ethylene glycol) extended urethane dimethacrylate	PEG-U
Triethylene glycol dimethacrylate	TEGDMA

**Adhesive (multifunctional) monomers**	
Methacryloyloxyethyl phthalate	MEP
Zirconyl dimethacrylate	ZrDMA
Pyromellitic glycerol dimethacrylate	PMGDMA

**Components of polymerization initiating systems**	
Benzoyl peroxide	BPO
Camphorquinone	CQ
Diphenyl(2,4,6-trimethylbenzoyl) phosphine oxide & 2-hydroxy-2-methyl-1-phenyl-1-propanone	4265 Darocur
2,2′-Dihydroxyethyl-p-toluidine	DHEPT
Ethyl-4-N,N-dimethylamino benzoate	4EDMAB
Bis(2,6-dimethoxybenzoyl)-2,4,4-trimethylpentyl phosphine oxide & 1-hydroxycyclohexyl phenyl ketone	1850 Irgacure
2-benzyl-2-(dimethylamino)-1-(4-(4-morphollinyl)phenyl)-1-butanone	369 Irgacure

Light-cure (LC) resins were formulated by combining the selected base (mass fractions varying from 16.8% to 62.9%), diluent monomers (mass fractions varying from 10.0% to 52.3%) and adhesive monomers (mass fractions varying from 0.8% to 5%) and blending in the chosen visible light initiator system (CQ and 4EDMAB (a mass fraction of 0.2% and 0.8%, respectively), or 4265 Darocur (a mass fraction of 0.8%), 1850 Irgacure (a mass fraction of 1.0%) or 369 Irgacure (a mass fraction of 1.5%)) while magnetically stirring (38 rad/s) the mixture at room temperature under safe lighting until achieving a uniform consistency of the blend. Chemical-cure (CC) resins were prepared by initially combining the monomers and homogenizing the unactivated resin via magnetic stirring. The resin was then split into two parts to which the individual CC components (BPO and DHEPT; a mass fraction of 2.0% and 1.0%, respectively) were added separately, and each mixture was stirred magnetically at room temperature until fully homogenized. Dual-cure ((DC); *i.e.*, the combined light and chemical cure) resins required, similarly to CC resins, a preparation of a two separate batches of the same resin which were light activated (1850 Irgacure) and contained either BPO or DHEPT.

The physicochemical characterization of the unfilled resins (copolymers) typically included determination of the degree of vinyl conversion (DVC), biaxial flexure strength (BFS) and water sorption (WS). The DVC was determined by using mid-FTIR or near-IR (NIR) spectroscopy. DVC values determined by mid-FTIR method were calculated from the reduction in the 1637 cm^−1^ absorption band for the vinyl group against that of an unchanged aromatic peak (1538 cm^−1^; internal standard) [35,36]. DVC values determined by NIR method [47] were calculated from the% change in the integrated peak area of the 6165 cm^−1^ methacrylate =CH_2_ absorption band between the polymer and monomer specimen. Spectra data were acquired before cure and 24 h after cure by collecting 64 scans at 2 wave-number resolution. Use of an internal reference was not required for the NIR measurements, provided that the thickness of monomer and polymer sample was measured. The BFS values of dry (24 h in the air at 23 °C) and wet (immersion in HEPES-buffered, pH = 7.4, saline solutions at 23 °C for time periods up to six months) copolymer disk specimens were determined using a piston-on-three-ball loading cell and a computer-controlled Universal Testing Machine (Instron 5500R, Instron Corp., Canton, MA, USA) operated by Testworks 4 software. The BFS values were calculated according to the ASTM specification [48]. To determine the WS of copolymer specimens, they were first dried over anhydrous CaSO_4_ until a constant mass was achieved (±0.1 mg) and then immersed in saline solutions (as in the BFS measurements). The mass of dry-tissue padded specimens recorded at different time intervals were used to calculate the WS of individual specimens (expressed as a% mass fraction) using the equation:

(1) $$\text{WS}=[({\text{W}}_{\text{t}}-{\text{W}}_{\text{o}})/{\text{W}}_{\text{o}}]\times 100$$

where W_t_ represents the sample mass at the time t, and W_o_ is the initial mass of dry sample.

### 2.3. Fabrication and Physicochemical Evaluation of Experimental ACP Composites

Composite pastes were made by combining by hand spatulation mass fractions of 60% resins and 40% ACP. Homogenized pastes were kept under a moderate vacuum (2.7 kPa) overnight to eliminate the air entrained during mixing. To make LC composite disk specimens, composite paste was packed into Teflon molds ((15.0 ± 0.5) mm in diameter and (1.5 ± 0.2) mm in thickness), each opening of the mold was then covered with Mylar film and a glass slide, and the assembly clamped in place by spring clips. The clamped specimens were photo-polymerized by irradiating sequentially each side of the mold assembly for 60 s with visible light (Triad 2000, Dentsply International, York, PA, USA). To prepare CC disk specimens, the BPO-containing paste and the DHEPT-containing paste were first combined in 1:1 mass ratio, and then packed into the molds where chemically-initiated polymerization occurred at room temperature. The DC specimens were prepared by combining the CC and LC procedure. All specimens were stored for 24 h in air at 23 °C before testing. The procedures identical to those used for composite disk specimen preparations were employed in fabricating copolymer specimens. Whenever the commercial materials were used as controls, their specimens were made by strictly following the manufacturer-recommended curing protocols.

In addition to the tests routinely performed with copolymer specimens (DVC, BFS and WS determinations (Section 2.2.), the composite specimens were mapped by FTIR-m, assessed for polymerization shrinkage (PS), polymerization shrinkage stress (PSS), shear bond strength (SBS) to dentin and the kinetics of the release of remineralizing ions into the immersion medium. While the DVC, PS and PSS tests were performed in the dry state, all other measurements were performed on both dry specimens and after their exposure to the aqueous environment. The extent of conversion of ACP fillers upon aqueous immersion was conveniently assessed by XRD.

**Figure 1:**
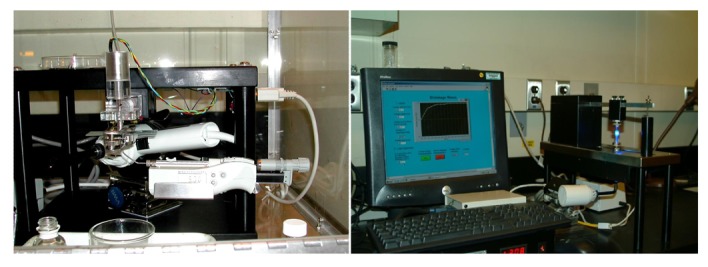
Photo-images of dilatometer (left) and tensometer (right) employed in polymerization shrinkage (PS) and polymerization shrinkage stress (PSS) studies, respectively.

The distribution of organic matrix and the inorganic filler on the surface of composite disks or the specimens' cross-sections was determined by FTIR-m (a Nicolet Magna-IR 550 FTIR spectrophotometer equipped with a video camera, a liquid nitrogen cooled-mercury cadmium telluride detector, a computerized, motorized mapping stage and the Omnic^®^ Atlus™ software, Spectra-Tech Inc., Shelton, CT, USA) [49]. The PS of composites was measured by a computer-controlled mercury dilatometer (Figure 1 (left); Paffenbarger Research Center (PRC), American Dental Association Foundation (ADAF), Gaithersburg, MD, USA) [45]. Composite pastes were cured using a standard (60 s + 30 s) light-cure exposure and data acquisition performed with (60 min + 30 min) regimens. The PS of the composites (expressed as a volume fraction, %) was calculated based on the known mass of the sample (50–100 mg) and its density (Sartorius YDK01 Density Determination Kit; Sartorius, Gőttingen, Germany). The originally constructed tensometer (PRC-ADAF, Gaithersburg, MD, USA) based on the cantilever beam deflection theory and the improved device (Figure 1 (right)) using the same principle—currently being tested in the Biomaterials Group, Polymers Division, National Institute of Standards and Technology (NIST; Gaithersburg, MD, USA)—were utilized to measure PSS (expressed in MPa) developing in the experimental composites. Detailed description of the PSS methodology is provided in [50]. The SBS of the ACP's composites to dentin through an acid-etched adhesive substrate was tested on extracted human molars whose roots were embedded in polycarbonate holders with a CC poly(methylmethacrylate) tray resin. The testing assembly and the bonding protocol that included successive coats of dentin adhesive and the experimental composite to dentin surfaces which were then light-cured, and the subsequent application of a commercial LC resin-based composite are described in detail in [51]. SBS specimens were debonded at a crosshead speed of 0.5 mm/min by (Universal testing machine as described in subsection 2.2). To assess the nature of the failures, debonded specimens were examined by optical microscopy (Leica MZ16 Optical Stereomicroscope, Wetzlar, Germany). In addition, the surface areas of different failure modes were measured by imaging software (ImageJ, National Institutes of Health, Bethesda, MD, USA; [52]).

Mineral ion release from the individual composite disk specimens was examined at 23 °C, in continuously stirred HEPES-buffered (Ph = 7.4) saline solutions (100 mL). Kinetic changes in the solution calcium and phosphate levels were determined by atomic emission spectroscopy (Prodigy High Dispersion ICP-OES, Teledyne Leeman Labs, Hudson, NH, USA).

### 2.4. Leachability of Unreacted Monomers from Copolymers and ACP Composites

**Figure 2:**
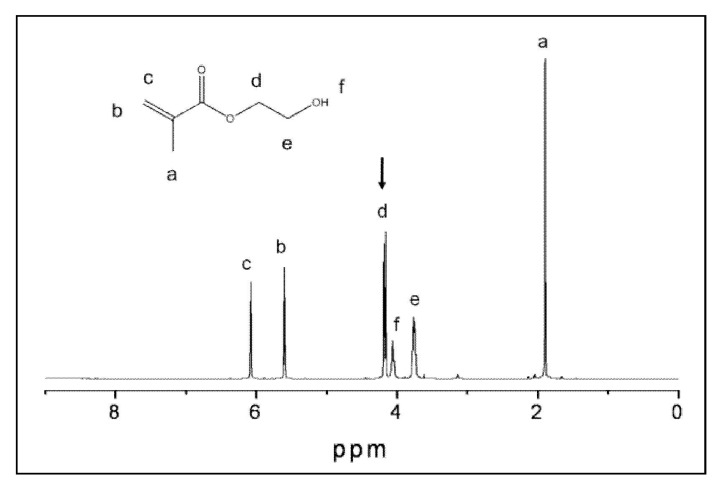
Nuclear magnetic resonance (^1^H NMR) spectrum of 2-hydroxyethyl methacrylate (HEMA) in acetone-*d_6_*. Peak integration values based on peak d at 4.22 ppm, representing two –CH_2_ protons.

The leachability of unreacted monomers and/or components of the initiator system from copolymer and composite specimens was assessed as follows: Individual copolymer (initial mass: (190.3 ± 15.5) mg) and composite specimens (initial mass: (237.4 ± 5.4) mg) were fully immersed in (28.0 ± 2.0) mL butylated hydroxyl toluene (BHT)-containing acetone (ACS purity; Fisher Scientific, Fair Lawn, NJ, USA) in tightly closed containers for 7 days at 23 °C with continuous magnetic stirring (32 rad/s). BHT was added to acetone at 0.01 mass % to prevent secondary polymerization in the extracts. Specimens (3/experimental group) were then removed from the extraction solution, blotted dry, and kept for 2 h in the hood to evaporate acetone. Each specimen was then kept under vacuum (approx. 90 kPa) for 7 d at 90 °C (temperature exceeded boiling point of acetone (56.5 °C)) to remove the absorbed acetone, left to cool to room temperature and its mass was recorded. The difference between the initial dry mass and the mass after the complete solvent removal corresponded to acetone-extracted leachables. The individual extracts, *i.e.*, acetone solutions after the removal of the disk specimens, were kept refrigerated in tightly sealed containers until use for ^1^H nuclear magnetic resonance (NMR) analyses. The ^1^H NMR spectra (JEOL GSX 270 MHz Fourier transform NMR spectrometer in acetone-*d_6_* (99.9 atom % D, containing 0.03% v/v tetramethyl silane; Sigma-Aldrich Co., USA) were collected on each sample and the peaks of interest for each component were integrated using BHT as an internal standard. The initial integration was performed separately for each monomer, the proton counts were confirmed based on the individual molecular structure, and these values were then used to determine leachable content of the extracts. Initiators (CQ and 4EDMAB) and the inhibitor BHT were also analyzed to confirm that there was no interference with the monomer peaks of interest. The differentiating peaks representing each component were integrated based on two -CH_2_ protons peak value for HEMA at 4.22 ppm (Figure 2). The integration values were used to calculate mol-% values of the individual components and, ultimately, their mass-% loss. To test the method, monomer samples of varying concentrations were run, integrated, and calculated. The NMR values *versus* calculated values for all samples showed minimal difference with a variation of (1.0 ± 0.3)%.

### 2.5. I n Vitro Cytotoxicity of Copolymers and ACP Composites

Cellular studies included phase contrast microscopy and Wst-1 [53] or MTT [54] assays for dehydrogenase activity. Osteoblast-like MC3T3-E1 cells (Riken Cell Bank, Hirosaka, Japan) were maintained in α-modification of Eagle's minimum essential medium (Biowhittaker, Walkerville, MD, USA) with a volume fraction of 10% fetal bovine serum (Gibco-BRL-Life Technologies, Rockville, MD, USA) and 60 mg/L kanamycin sulfate (Sigma, St Louis, MO, USA) in a fully humidified atmosphere with a volume fraction of 5% CO_2_ at 37 °C. The medium was changed twice a week. Cultures were passaged with EDTA-containing (1 mmol/L) trypsin solution (mass fraction of 0.25%; Gibco, Rockville, MD, USA) once a week. Specimen disks (5.3 ± 0.1) mm in diameter and (3.1 ± 0.1) mm in thickness) were first sterilized with 70% ethanol prior to extraction experiments. Each disk was then washed with 2 mL of media for 1 h and then fresh media was placed on each disk for 1 day extraction in the cell incubator at 37 °C. In parallel, a flask of 80% confluent MC3T3-E1 cells was passaged, seeded into well plates with 10,000 cells per well in 2 mL of media, and then placed in the incubator overnight. On the second day of the experiment, the medium from each “cell well” was removed and replaced with the 2 mL of extraction medium from one of the disk specimens (or with the positive or negative control media). The cells were incubated in the extracts, photographed (digital photography using an inverted phase contrast microscope, Nikon TE300, Melville, NY, USA) and then prepared for the Wst-1 or MTT assays. The Wst-1 tests were performed according to the following protocol: Extract-cultured cells and the controls without cells were combined with a Wst-1 (2-(4-iodophenyl)-3-(4-nitophenyl)-5-(2,4-disulfophenyl)-2H-tetrazolium, monosodium salt; Dojindo, Gaithersburg, MD, USA) solution in HEPES buffer, individually added to wells and incubated for 2 h at 37 °C. Aliquots from each well were transferred to a well-plate and absorbance was read at 450 nm with a plate-reader (Wallac 1420 Victor^2^, Perkin Elmer Life Sciences, Gaithersburg, MD, USA). The MTT assays were performed as follows: cells cultured in the extracts were rinsed with 1 mL phosphate buffered saline solution (PBS; 140 mmol/L NaCl, 0.34 mmol/L Na_2_HPO_4_, 2.9 mmol/L KCl, 10 mmol/L HEPES, 12 mmol/L NaHCO_3_, 5 mmol/L glucose, pH = 7.4) and 0.125 mL/well of 3-(4,5-dimethylthiazol-2-yl)-2,5-diphenyltetrazolium bromide (MTT) solution (5 mg/mL MTT in PBS). After 2 h incubation at 37 °C, the MTT solution was removed, the insoluble formazan crystals were dissolved in 0.1 mL dimethylsulfoxide (DMSO), and the absorbance was measured at 540 nm with a plate reader. The blank values (the well that contain only the PBS, MMT and DMSO solutions) were subtracted from each of the experimental values as background.

### 2.6. Statistical Methodology

Graphical data analysis and analysis of variance (ANOVA) were performed to evaluate the experimental data as a function of composite makeup, storage times or any other relevant factor involved in the experimental design. For the cases where the overall statistically significant effects are found with ANOVA, further tests were performed to determine the significant differences between the specific groups using a multiple comparison procedure. All tests were 2-sided at α = 0.05. Statistical analyses of the data were done by means of Microsoft Office Excel 2007, or SigmaStat version 2.03 (SPSS Inc., Chicago, IL, USA). Typically, one standard deviation (SD) is identified in this paper for comparative purposes as the estimated uncertainty of the measurements.

## 3. Results and Discussion

### 3.1. Effects of Precipitating Conditions and Treatments on Properties of ACP Fillers

The excessive and uncontrolled agglomeration of ACP particles during ACP synthesis typically yields highly clustered ACP solids that disperse non-uniformly in the resin phase of the composites [35,46,49]. Consequently, ACP polymeric composites are mechanically inferior to surface-treated, glass-reinforced resin materials [36,44]. Moreover, the state of ACP agglomeration also affects the ion release from the polymeric ACP composites [46]. It has been documented that various additives (inorganic salts, organic molecules with various functional groups, polymeric molecules and polyelectrolytes), introduced *ab initio* during the synthesis, affect the kinetics of the spontaneous precipitation of CaPs from supersaturated solutions and determine both the type and stability of precursor phases [1,2,3,55,56]. We have performed a series of ACP syntheses with the specific aim to assess whether the introduction of: (1) cations [monovalent silver (Ag^+^); divalent iron (Fe^2+^) and zinc (Zn^2+^); trivalent iron (Fe^3+^) and alumina (Al^3+^); and tetravalent silica (Si^4+^) or zirconia (Zr^4+^)]; (2) surfactants [nonionic (Triton 100, Tween 80 and Zonyl FSN); or anionic (Zonyl FSP)]; and (3) polymers [poly(ethylene oxide) (PEO; molecular mass, M_w_ = 8 K, 100K or 1000K) during the synthesis can reduce the extent of ACP agglomeration (and, as a consequence of the reduced ACP's particle, size improve the mechanical performance of the experimental composites formulated from such modified ACPs). Surface modification of ACP with commonly utilized silane agents, APTMS and MPTMS, as well as grinding [45] and mechanical milling [46] were proposed and explored as alternative ways to break up large ACP agglomerates. It was assumed that neither of the above treatments would significantly compromise ACP's bioactivity as remineralizing (calcium and phosphate ion releasing) agent. To test whether the attained levels of the mineralizing ions released from the experimental composites formulated with am-, g- and m-ACP were adequate to create an environment favorable for augmenting mineral-deficient tooth structures (ΔG^0^ values < 0), the thermodynamic stability of the immersion solutions was calculated with respect to stoichiometric HAP using the Gibbs free-energy expression [35,36,44]:

(2) $$\Delta {\text{G}}^{0}=-2.303\left(\text{RT}/\text{n}\right)\;\ln\left(\text{IAP}/{\text{K}}_{\text{sp}}\right)$$

where IAP is the ion activity product for HAP, K_sp_ is the corresponding thermodynamic solubility product, R is the ideal gas constant, T is the absolute temperature, and n is the number of ions in the IAP (n = 18).

**Figure 3:**
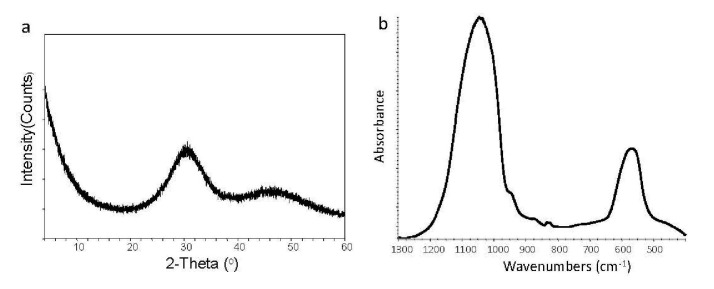
X-ray diffraction (XRD) pattern (**a**) and Fourier-transform infrared (FTIR) (**b**) spectrum representative of ACP.

A typical XRD pattern (Figure 3(a)) and the corresponding FTIR spectrum (Figure 3(b)) of any uncoverted ACP (independent of the type of additive, silanization treatment, grinding or milling) revealed lack of crystalline regularity, *i.e.*, the ACP's striking feature that distinguishes it from other CaPs [1,2,3,4]. The XRD diffraction analysis showed only two diffuse broad bands in the 2θ = (4 to 60)° region. Such pattern is indicative of a solid in which no translational and orientational long-range order of the atomic positions could be detected [1]. The corresponding FTIR spectrum consisted of two wide PO_4_ absorbance bands at (1200 to 900) cm^−1^ and (630 to 550) cm^−1^, typical for phosphate stretching and phosphate bending, respectively.

Irrespective of the treatment, all ACPs had heterogeneous PSDs with particle diameters (expressed as the equivalent spherical diameter) spanning from submicron values up to 200 μm or more in diameter. The heterogeneity of the particle sizes was confirmed by SEM observations (images not shown). For both Fe^2+^- and Fe^3+^-ACP, signs of conversion to crystalline apatite were seen in their XRD and FTIR scans. In addition, significant color changes occurred in Ag-, Fe^2+^- and Fe^3+^-solids. This unwanted color change was due to the co-precipitation of light–sensitive Ag- and colored Fe-phosphates with ACP. Because of the color instability and accelerated conversion to apatite, these cation-ACPs were not further evaluated. In the non-redox sensitive cation series, the median particle diameter (d_m_) of Zn-ACP and Al-ACP was significantly lower than the d_m_ of Si-ACP and Zr-ACP. PEO-ACPs had significantly higher d_m_ compared to all cation-ACPs or no-additive, control ACP. Observed differences in the mean values of d_m_ between different cations would suggest slight modifications in the degree of ACP's agglomeration, which appear random rather than systematically related to ionic potential of cations. The apparently higher extent of PEO-ACP agglomeration could possibly be attributed to a mechanism similar to “polymer bridging”, that reportedly controls the agglomeration of apatite particles in the presence of high-molecular mass polymers [56]. The average water content (TGA results) of the modified ACPs (mass fraction of 15.7%) appeared unaffected by the type of additive used during the synthesis. It corresponded to approximately 2.6 water molecules per structural ACP unit with roughly 2/3 of this water being surface-bound (weight loss below 130 °C) and the remaining 1/3 being structurally incorporated (weight loss at (130 to 600) °C)). Water content of all ACPs correlated well with the values reported in the literature (mass fraction of up to 18% [1,57]. Apparently, no correlation existed between the ACP particle size and the BFS of composites in either dry or wet state. The highest dry BFS value was attained in PEO-ACP composite group (76.0 MPa). Dry BFS values of the cation-ACP specimens ranging from 41.5 MPa to 54.3 MPa were not statistically different from the unmodified ACP control (56.0 MPa). After exposure to aqueous environment, the BFS of PEO-ACP composites was reduced by 69% compared to their dry values. In cation-ACP series, the extent of BFS reduction in going from dry to wet state was nonexistent (Zn-ACP) or ranged from 10% (Zr-ACP) to 41% (Al-ACP). The same reduction in the control (no additive) ACP group was 29%.

The MPTMS-silanized ACP formulated into EBPADMA/TEGDMA/HEMA/MEP (ETHM resin)-based composites showed improved biaxial flexure strength (BFS) compared to the unsilanized ACP/ETHM composites and APTMS-ACP/ETHM composites. In dry state, MPTMS-ACP/ETHM composites attained a high BFS ((73.3 ± 7.5) MPa) compared to APTMS-ACP ((46.4 ± 9.8) MPa) and am-ACP control composites ((47.6 ± 7.5) MPa) [36]. More importantly, even upon soaking, the MPTMS-ACP/ETHM composites were still over 50% stronger than the control composites suggesting that MPTMS could potentially ameliorate the overall plasticization by water of composites. Therefore, utilizing MPTMS-ACP as bioactive filler should be considered when designing remineralizing composites for applications that require enhanced mechanical stability.

Results of ACP surface-modification studies with selected cations, surfactants and polymers do not rule out the possibility that other additives and/or different surface treatments could be more effective in homogenizing the PSD of ACP fillers, more uniformly dispersing ACP within resin matrices and improving the mechanical stability of the composites.

**Figure 4:**
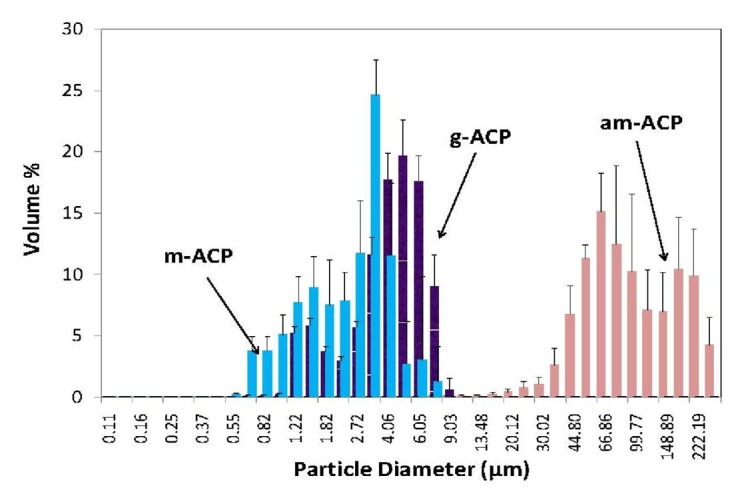
Volume size distribution histograms of as made (am-), ground (g-) and milled (m-) Zr-ACP dispersed in isopropanol. Indicated are mean values + standard deviation of three repetitive runs in each group.

Since only Zn- and Zr-ACP showed an increase in strength after immersion compared to the composites formulated with the unmodified ACP filler, and since Zr-ACP was also shown [58] to maintain a desired remineralizing potential for prolonged time periods, grinding and milling experiments were performed with Zr-ACP. The resulting volume size distribution histograms are shown in Figure 4. These PSDs indicate that the volume fraction of fine particles increases in following order: m-ACP ≫ g-ACP ≥ am-ACP. As a result, blending of g- and, particularly, m-ACP into the resin was much easier and took less time than the same process using am-ACP. Moreover, at the same filler level, pastes with g- and m-ACP were more flowable compared to the am-ACP composite paste (typically very viscous and non-flowable). The narrower PSD obtained through grinding and, especially, milling apparently improved dispersion of these fillers within the matrices and, in turn, the mechanical properties of g- and m-ACP composites (Table 4). More homogeneous dispersion of g- and m-ACP fillers throughout the composites, *i.e.,* a lesser number of voids/defects existing throughout the bodies of the composite disk specimens, resulted in reduced WS in g- and m-ACP composites. While the levels of the mineralizing ions released into buffered saline environment from g- and m-ACP composites and the resulting supersaturations with respect to HAP were somewhat reduced compared to am-ACP composites, the conditions favorable for regenerating mineral-deficient tooth structures (ΔG^0^ values ≪ 0) were maintained in all systems.

**Table 4:** Median diameter (d_m_) of the am-, g- and m-Zr-ACP fillers, and the biaxial flexural strength (BFS; after 1 mo aqueous immersion), maximum water sorption (WS_max_) at 75% relative humidity and the anti-demineralizing/remineralizing capacity (expressed as the thermodynamic stability of the solutions with respect to stoichiometric HAP; ΔG^0^) of their EBPADMA/TEGDMA/HEMA/MEP resin composites. Indicated are mean values + one standard deviation (n ≥ 5).

**Parameter**	**am-ACP**	**g-ACP**	**m-ACP**
d_m_ (μm)	80.0 ± 4.7	4.5 ± 0.8	3.3 ± 0.5
BFS (MPa)	42.2 ± 6.7	50.0 ± 8.0	56.4 ± 7.7
WS_max_ (mass%)	3.1 ± 0.4	2.5 ± 0.5	1.7 ± 0.2
ΔG^0^ (kJ/mol)	−(5.7 ± 0.2)	−(4.9 ± 0.6)	−(5.1 ± 0.3)

In conclusion, grinding and/or milling are identified as simple yet effective approaches for reducing the size and number of large am-ACP agglomerates. Alterations in the milling and/or grinding regimens may be required to additionally enhance the mechanical interlocking at the filler/matrix interfaces which should boost the mechanical performance of composites. Due to the fact that a significant level of de-agglomeration can be achieved with either grinding or milling, am-Zr-ACP was used as a standard ACP filler in all subsequent studies described in this manuscript.

### 3.2. Structure/Composition/Property Relationships in Experimental Resins and Their ACP Composites

Typically dental resins contain a relatively viscous base monomer which minimizes polymerization shrinkage by virtue of its relatively large molecular volume and can also enhance the modulus of the cured polymer due to its relatively rigid structure, and a diluent co-monomer which reduces resin viscosity and improves handling properties and overall vinyl conversion due to its smaller molecular volume and greater flexibility [35]. The most commonly utilized copolymers are based on the base monomer Bis-GMA and the diluent monomer TEGDMA. The hydroxyl groups of Bis-GMA and the ethylene oxide segments of TEGDMA contribute to the relatively high water sorption (WS) of Bis-GMA/TEGDMA copolymers [36]. High concentrations of the more rigid ring structure of Bis-GMA typically result in monomer systems with relatively low degrees of vinyl conversion (DVC) and low polymerization shrinkage (PS). The relatively low cure efficiency at ambient temperatures and subsequent plasticization of Bis-GMA/TEGDMA copolymers by oral fluids (primarily water) affect the service life of these composites. Alternative base monomers and/or diluent monomers have been explored to overcome some of the known shortcomings of the Bis-GMA/TEGDMA copolymers. Dental polymers based on EBPADMA, a more hydrophobic analog of Bis-GMA with a higher molecular mass but with a more flexible structure and lower viscosity, reportedly show higher DVC and lower PS than Bis-GMA/TEGDMA resins [44]. In photo-polymerization, the UDMA monomer has been shown to be more reactive than Bis-GMA or EBPADMA [47].

In our studies, series of resins containing Bis-GMA, EBPADMA or UDMA as base monomers were formulated with TEGDMA, HmDMA, poly(ethylene glycol)-extended UDMA (PEG-U) and HEMA as diluent co-monomers. Surface active monomers ZrDMA or MEP were used as monomeric additives in an attempt to promote adhesion to ACP. Introduction of ZrDMA and MEP into resin matrix was motivated by the ability of these monomers to improve coupling between the organic phase and the inorganic ACP filler (ZrDMA) and possibly also enhance adhesion of the composites to tooth structures (MEP) [35,36,44]. It is hypothesized that by appropriate resin modification an optimal viscosity suitable for the incorporation of particulate ACP filler can be achieved and satisfactory levels of DVC attained. It is also postulated that the experimental ACP composites will absorb enough water to release mineral ions at levels needed for tooth mineral recovery without seriously compromising the mechanical stability of the composites. To test these hypotheses, an extensive physicochemical evaluation of both the unfilled resins (copolymers) and their ACP composites was performed.

The results of physicochemical screening and the mechanical testing of the series of binary and ternary experimental resins based on Bis-GMA, EBPADMA and UDMA and their corresponding am-ACP composites formulated for intended applications as dental sealants and/or base/liners are compiled in Table 5. LC Bis-GMA- and EBPADMA-based copolymers and composites, and to a lesser extent UDMA-based copolymers and their composites, achieved high DVC values (on average (80.8 to 90.8)%). Regardless of the resin matrix composition, the DVCs of Bis-GMA- and EBPADMA-based ACP composites were generally lower than DVC values attained in their UDMA-based counterparts. This fact can be explained by the higher reactivity of UDMA monomer in comparison with Bis-GMA or EBPADMA [47]. Typically, higher DVC values were attained in all resins with relatively high contents of HEMA (≥28 mass %). This effect is related to HEMA's high diffusivity and mono-functionality. Expectedly, the composites that yielded DVCs between 80.8% and 86.7% also showed relatively high PS (on average, between 6.4 vol % and 7.4 vol % for all experimental groups) that exceeded the PS values typically reported for highly glass-filled commercial composites (1.9 vol % to 4.1 vol %), and the ACP composites fell into the category of either flowable composites or adhesive resins (reported PS values (3.6 to 6.0) vol % and (6.7 to 13.5) vol %, respectively [59,60]. These high PS values can be attributed in part to a filler level of only 40 mass % in ACP experimental materials compared to that of up to 85% of silica-based fillers in conventional composites. Based on PS results alone, the inclusion of bulkier but relatively low viscosity resins or ring-opening monomers [61,62] into experimental resins and/or higher contents of ACP may be required to obtain composites with lower PS while maintaining satisfactory DVC.

**Table 5:** Physicochemical characteristics (degree of vinyl conversion (DVC) (24 h post-LC), WS_max_ (at 75% relative humidity (RH)), PS, BFS (dry and wet specimens after 1 mo of aqueous immersion)) of Bis-GMA-, EBPADMA- and UDMA-based copolymers and their ACP composites, and the supersaturation of the immersing solutions (ΔG^0^) with respect to stoichiometric HA (calculated from ion release data). Diluent monomers: HEMA, HmDMA and TEGDMA; photoinititor system: CQ and 4EDMAB.Indicated are mean values ± standard deviation. Number of repetitive experiments in each group, n = 4 (ΔG^0^; WS), n ≥ 4 (BFS), n ≥ 8 (DVC) and n ≥ 9 (PS). nd–not determined; n/a–not applicable.

**Base monomer**		**DVC** (%)	**WSmax** (mass %)	**PS** (vol %)	**BFS** (MPa)		**ΔG^0^** (kJ/mol)

					Dry	Wet	

Bis-GMA	copolymer	88.6 ± 2.7	3.4 ± 0.3	nd	136 ± 35	131 ± 34	n/a
	composite	80.8 ± 4.6	2.8 ± 0.2	6.4 ± 1.5	61 ± 12	53 ± 11	−[5.96 ± 0.12]

EBPADMA	copolymer	90.8 ± 1.6	2.0 ± 0.3	nd	116 ± 22	117 ± 34	n/a
	composite	81.2 ± 3.6	2.6 ± 0.2	7.4 ± 1.1	59 ± 9	53 ± 9	−[7.23 ± 0.23]

UDMA	copolymer	87.9 ± 1.7	2.6 ± 0.3	nd	167 ± 33	125 ± 35	n/a
	composite	86.7 ± 2.0	2.8 ± 0.1	6.7 ± 0.9	61 ± 9	46 ± 12	−[5.35 ± 0.17]

Water uptake, especially if excessive, generally causes a decrease in mechanical strength, depression of the glass transition temperature due to plasticization, solvation, reversible rupture of weak inter-chain bonds and/or irreversible disruption of the polymer matrix [63,64]. In the case of ACP polymeric composites, the overall WS profiles are additionally affected by ACP-water interactions, and WS regulates the kinetics of the intra-composite ACP to HAP conversion. Ultimately, WS determines the remineralizing capacity of bioactive ACP composites. WS_max_ values for all resins and their ACP composites were reached within two weeks of saline immersion. They were generally highest for ternary formulations containing TEGDMA and HEMA, and lowest for binary resins that contained the relatively hydrophobic HmDMA. These differences are related to the relative portion of hydrophilic (HEMA and TEGDMA) or hydrophobic (HmDMA) monomer in the matrix which, ultimately, determines the overall hydrophilicity/hydrophobicity balance of the resin.

When exposed to aqueous milieu, ACP composites formulated with either of the experimental resins, released calcium and phosphate ions at such levels that the immersion solutions became highly supersaturated with respect to HAP (ΔG^0^ ≪ 0). Since only marginal differences were seen in the WS of Bis-GMA-, EBPADMA- and UDMA-based copolymers, the observed trend of decreasing ΔG^0^, *i.e.*, ΔG^0^_EBPADMA_ > (ΔG^0^_Bis-GMA_ ≥ ΔG^0^_UDMA_) is attributed to differences in the chemical structure and the composition of the monomer systems. Generally, more negative ΔG^0^ values (higher supersaturations; higher remineralizing potential) attained in systems containing EBPADMA as a base monomer and HEMA as a co-monomer were attributed to a more open cross-linked network structure of the resin matrix (EBPADMA), and the increased internal mineral saturation that allows uptake of more water and/or better accessibility of ACP filler to the water already entrained in the HEMA-rich resins.

**Figure 5:**
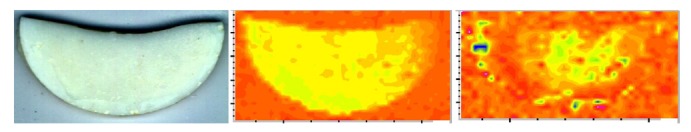
Micro-FTIR data (visual image (left), CO (carbonyl) map (middle), PO_4_ map (right)) illustrating a typical, highly heterogeneous distribution of am-ACP on the surface of Bis-GMA/TEGDMA/HEMA composite disk specimen (used in BFS measurement; specimen diameter = 15 mm). Color code: CO and PO_4_ peak intensities decreased as follows: blue > green > yellow > orange > red.

In the copolymer series, Bis-GMA- and EBPADMA-based copolymers did not weaken upon immersion while UDMA-based copolymers deteriorated upon exposure to an aqueous environment (their wet *vs.* dry BFS was reduced on average 25%). Generally, dry ACP composites had substantially lower BFS than the corresponding copolymers regardless of the resin composition. This reduction in BFS values in going from dry copolymer to composite was between 49% (EBPADMA) and 63% (UDMA). The strength of all but binary, HmDMA-containing composites diminished further upon soaking. This decline in BFS of dry *vs.* wet composites ranged from 10% (EBPADMA) to 25% (UDMA). The existence of numerous defects/voids (resin-rich, ACP-depleted regions) and the random distribution of large am-ACP agglomerates, typically seen in these composites (Figure 5; [49]), is responsible for inadequate filler/resin interlocking and the mechanical instability of these materials.

Properties of the am- and m-ACP/EBPADMA/HEMA/MEP/TEGDMA (assigned ETHM resin) composites formulated for orthodontic application are compared in Table 6. Two series of resins were examined: series (1) with the molar ratio of EBPADMA/TEGDMA from 0.13 to 0.50 and a constant molar ratio of HEMA/MEP of 4.28; and series (2) with the molar ratio of EBPADMA/TEGDMA from 0.50 to 1.33 and a constant molar ratio HEMA/MEP of 8.26.

**Table 6:** DVC (24 h post-LC), WS_max_ (at 75% RH), PS, BFS (wet specimens after 1 mo immersion) and thermodynamic stability of the immersing solutions (ΔG^0^) attained with am-ACP/ETHM composites (series 1), and am-and m-ACP/ETHM composites (series 2). Indicated are mean values ± standard deviation for n ≥ 6 (DVC), n ≥ 3 (PS), n = 5 (WS, BFS) and n = 4 (ΔG^0^). Nd—not determined.

**ETHM resin**		**DVC** (%)	**WS_max_** (mass %)	**PS** (vol %)	**BFS** (MPa)	**ΔG^0^** (kJ/mol)

**Series 1**	am-ACP	84.8 ± 6.5	3.5 ± 0.5	6.9 ± 0.6	44.5 ± 8.2	−[4.65 ± 0.31]

**Series 2**	am-ACP	72.2 ± 3.8	2.5 ± 0.3	nd	36.1 ± 6.7	−[4.56 ± 0.23]
	m-ACP	76.7 ± 3.9	1.8 ± 0.2	nd	56.5 ± 9.4	−[4.18 ± 0.39]

In series 1, variations in resin composition did not significantly affect the DVC, WS_max_, PS, BFS or ΔG^0^ of am-ACP/ETHM composites. However, in series 2, both am- and m-ACP/ETHM composites attained lower DVCs and showed less affinity for WS compared to am-ACP counterparts in series 1. Apparently lower BFS of am-ACP composites in series 2 (on average (36.1 ± 6.7) MPa) compared to series 1 (on average (44.5 ± 8.2) MPa) was not statistically significant. The m-ACP composites retained higher BFS upon soaking (on average (56.5 ± 9.4) MPa) compared to am-ACP formulations. This improved strength and lower WS of m-ACP composites is related to the improved dispersion of milled ACP filler throughout the composites compared to the coarse, am-ACP. This lower WS did not significantly affect the overall remineralizing potential of m-ACP composites.

The results of the physicochemical evaluation of the experimental endodontic sealers formulated with UDMA/PEG-U/HEMA/MEP (UPHM) resin and am- and g-ACP fillers are presented in Table 7. At 24h post-cure, all LC UPHM copolymers attained exceptionally high DVC values. The DVC values obtained in chemically cured (CC) copolymers were, on average, 35% lower than DVC values achieved in LC formulations. In DC series, the attained DVCs were comparable (am-ACP composites) or even higher (g-ACP composites) than DVC of copolymers. Differences in DVC values attained in various LC formulations (for details, see subsection 2.2.) were not statistically significant. Based on these findings, CC composites were excluded from further testing. PS could be measured successfully only with LC composites. In DC system, however, hardening of the paste occurred within less than 10 min of mixing the chemically activated components, thus making the material unsuitable for PSS measurement by tensometry.

**Table 7:** Properties of light cure (LC) and dual cure (DC) experimental endodontic sealer {UPHM copolymers and their ACP composites (mean value ± standard deviation)}. Number of repetitive runs ≥ 3/experimental group. am—as made ACP; g—ground ACP; nd—not determined; n/a—not applicable.

**Property**	**LC-UPHM**			**DC-UPHM**		

	copolymer	composite		copolymer	composite	
	
		am-ACP	g-ACP		am-ACP	g-ACP
**DVC** (%)	95.7 ± 2.2	86.4 ± 1.9	88.4 ± 2.4	79.3 ± 3.4	76.0 ± 4.4	85.4 ± 3.2
**PS** (vol%)	n/d	7.1 ± 0.3	6.9 ± 0.1	n/d	n/d	n/d
**PSS** (MPa)	n/d	4.8 ± 0.2	4.1 ± 0.2	n/d	3.7 ± 0.3	3.6 ± 0.2
**WS_max_** (mass%) RH	3.2 ± 0.3	3.2 ± 0.2	3.2 ± 0.1	2.5 ± 0.5	2.9 ± 0.1	2.6 ± 0.2
immersion	6.7 ± 0.6	8.7 ± 0.4	9.4 ± 0.4	6.2 ± 0.5	7.4 ± 0.5	8.6 ± 0.4
**HE** (vol%)	5.4 ± 2.4	12.5 ± 1.8	11.8 ± 1.3	6.7 ± 1.7	13.0 ± 1.1	13.6 ± 1.7
**BFS** (MPa)	137.1 ± 24.9	39.4 ± 3.3	44.4 ± 4.4	124.3 ± 3.4	49.4 ± 9.4	47.3 ± 8.9
**ΔG^0^** (kJ/mol)	n/a	−[7.37 ± 0.33]	−[7.14 ± 0.46]	n/a	−[7.44 ± 0.39]	−[6.96 ± 0.32]

The mean PS values for LC am- and g-ACP UHMP composites (7.1 vol % and 6.9 vol %, respectively) were comparable to PS measured in Bis-GMA-, EBPADMA- and UDMA-based composites (Table 5: (6.4 to 7.4) vol%) and EHMT composites (Table 6: on average (6.9 ± 0.6) vol %). The relatively high PS of ACP/UPHM composites may be attributed to the intensified hydrogen bonding that is, generally, likely to occur in resin matrices with a relatively high amount of HEMA (16.8 to 17.3 mass %) and could ultimately lead to the densification of polymerization [65]. In LC UPHM composites, the polymerization shrinkage stress (PSS) decreased in going from am-ACP to g-ACP. PSS was generally lower in DC compared to LC UHMP composites. The PSS developed in LC am-ACP/UHMP formulations ((4.1 to 4.7) MPa) compares well with the PSS that developed in LC am-ACP/UDMA/HEMA composites (on average (4.5 ± 0.1) MPa [66]) suggesting that, in UPHM matrices, the stress originating from the PS is not elevated by the simultaneous inclusion of HEMA and PEG-U into the resin matrix. The relatively high PS seen in UPHM composites is likely to be compensated by the significant hygroscopic expansion (HE) of these materials (up to 13.6 vol %) due to water uptake upon aqueous immersion of the specimens. Beneficial aspects of HE have indeed been demonstrated by other researchers [67,68]. For the intended endodontic application, a material's mechanical strength is not the most critical property. The BFS values of the immersed specimens ranging from 39.4 MPa to 49.4 MPa are considered satisfactory for an endodontic sealer. It is more important that with respect to BFS, DC UPHM formulations were not inferior to their LC counterparts. Significantly, the BFS values across the four ACP/UPHM composite groups compare well with the BFS values of the experimental orthodontic adhesive (ACP/ETHM composites; Table 6), shown to efficiently restore subsurface carious lesions in human teeth [37]. The ion release levels attained with ACP/UPHM composites yielded solutions supersaturated with respect to stoichiometric apatite at levels equal to or higher than the remineralization potential of other experimental formulations (Table 5 and Table 6). It is, therefore, expected that the experimental ACP/UPHM composites would have strong potential to inhibit or possibly even reverse root caries in endodontic applications.

**Figure 6:**
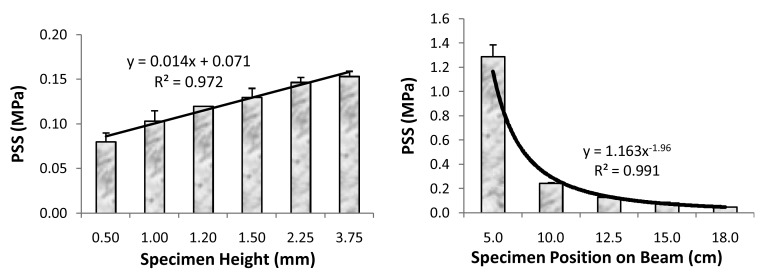
Correlation between the measured PSS values and the specimen height (left) and its position on cantilever beam (right). Indicated are mean values + one standard deviation for a minimum of three specimens per condition.

It is well known that the polymerization of methacrylate monomer systems is strongly affected by polymerization processing factors such as the type and concentration of initiators which determine reaction kinetics and DVC attained upon polymerization. Besides the type and concentration of initiators, other important material and processing factors that affect the PS, PSS and Young's modulus of composites are filler type and content, resin type and composition, and configuration or C-factor, *i.e.*, ratio of the bonded to the un-bonded (free) surface area of the composite in a cavity. Despite the numerous efforts, as reviewed in [69,70,71,72,73,74,75,76], the kinetics of both PS and PSS in these systems is still not fully understood. In an attempt to mimic constrained PS and PSS that occurs in composites bonded to tooth structure we have investigated whether larger unbounded surface area (lower C-factor) yields lower PSS values by allowing greater plastic deformation to occur during polymerization before the gel point is reached (as postulated in [76]). Using the cantilever beam tensometer, we assessed the effect of variations in C-factor on PSS in a typical am-ACP/Bis-GMA/TEGDMA remineralizing composite and a typical commercial glass-filled composite. The measured PSS values (PSS_meas_) were normalized for mass to specimens with a C-factor of 1.33 (height = 2.25 mm) as controls to give calculated PSS values (PSS_calc_). DVC attained in composites were measured by NIR spectroscopy. In both the experimental ACP composite and control composite, PSS_calc_ increased with the increasing C-factor, confirming the hypothesis that cavity configuration affects PSS values. Other reports on PSS developing in very thins layers of adhesive or composite have shown a substantial disparity in PSS *vs.* specimen thickness [77,78]. For the examined range of specimen thicknesses (0.50 to 3.75) mm, no correlation existed between the PSS_meas_ and the specimen thickness for both types of composites. One could possibly attribute such results to the insufficient sensitivity of the tensometer to detect differences in PSS for composite specimens over the range of C-factors studied. However, for a specimen thickness more akin to the resin-composite direct restorations (thickness (0.8 to 1.50) mm), it was also found that the variations in PSS are minimal and not affected by instrument compliance [79]. Higher PSS values for the experimental am-ACP/Bis-GMA/TEGDMA composite compared to the commercial control are not unexpected. The higher DVC attained in ACP composites lead to higher PS and PSS compared to a less converted and more highly filled control material. Also the greater translucency of the ACP composite may have enhanced its degree of radiance and contributed to its higher DVC. An additional critical factor is the composition of resin phase: Bis-GMA/TEGDMA (ACP composite) *vs.* UDMA-modified Bis-GMA/TEGDMA (commercial control). Urethane-modified Bis-GMA oligomer would be expected to shrink less than Bis-GMA/TEGDMA. With respect to PSS, high levels of filler in composite are desirable since their contribution to stress is usually minimal [80], but filler may also contribute to PSS by increasing elastic modulus. Other material factors being equal, the most rigid material, *i.e.*, the one with highest modulus will show the highest PSS. In a related study, performed with a new tensometer with a more slender cantilever beam, the objective was to validate the instrument's performance by measuring the PSS that develops as a function of: 1) variable height of the composite specimen at a fixed position on the beam, and 2) variable specimen's position on the beam. The measurements with a new tensometer also did not show any relationship with the C-factor of the composite specimen. However, a direct, linear correlation with specimen height (mass) was obtained (Figure 6 (left)). The PSS as a function of beam position was best described by an exponential equation indicative of an inverse relationship between the PSS and beam position (Figure 6 (right)) and indicating the effect of the instrument compliance on PSS. In view of the lack of correlation between PSS_meas_ and C-factor for composites, it may be prudent to investigate the temperature of the composites' exothermic polymerization, and cooling that follows, along with the PSS measurements [81]. Nevertheless, results of both studies strongly suggest that processing factors need to be considered when assessing PSS development in composites.

**Figure 7:**
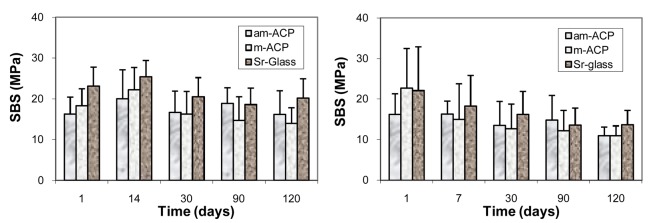
Shear bond strengths (SBS; mean value + one standard deviation) of am- and m-ACP composites formulated with **Bis-GMA**/TEGDMA/HEMA/ZrDMA resin (**left**) and **EBPADMA**/TEGDMA/HEMA/MEP resin (**right**) composites as a function of time of aqueous immersion. Number of tested specimens: n ≥ 7 (ACP/BTHZ) and n ≥ 5 (ACP/ETHM).

Shear bond strength (SBS) of the experimental base and lining composite and orthodontic composite formulated from LC Bis-GMA, TEGDMA, HEMA and ZrDMA (designated BTHZ) and EBPADMA, TEGDMA, HEMA and MEP (designated ETHM) resins, respectively, and am-ACP or m-ACP were compared with the SBS of corresponding composites made with a fluoride-releasing strontium glass (Sr-glass). Results of the SBS testing are compared in Figure 7.

The SBS values of the base and lining composites were inferior to SBS values of the Sr-glass composites only after 24 h immersion. The apparent differences in the mean SBS values between different filler groups at longer time intervals were not statistically significant. In the orthodontic series, only initially (at 24 h), m-ACP composites showed stronger bonding than am-ACP composites. With water aging, however, similarly to the base and lining composites, bond strengths attained with all three composite types became statistically indistinguishable. Following the six months of immersion, the overall reduction in the SBS was 42%. The results of this study suggest that, despite the more numerous flaws that am-ACP filler may introduce into the microstructure of composites compared to m-ACP and, especially to Sr-glass control, there was no detectable adverse affect on the short- and mid-term bonding behavior to these materials and both, am- as well as m-ACP composites formulated with either experimental resin (BTHZ or ETHM) performed at least as well as Sr-glass-reinforced composites, while providing an additional bioactive component that may prevent and/or delay demineralization and promote remineralization of tooth enamel. Based on the SBS results, both types of the experimental ACP composites appear to be suitable for the intended applications as remineralizing base/liner or orthodontic cement. The in-depth analyses of the failure modalities reported in [52,82], however, suggests that, despite the indistinguishable differences in the measured bonds strength compared to am-ACP, m-ACP composites may offer greater potential in clinical applications. To improve the clinical appeal of these experimental composites, further work on designing adhesive resins with improved long-term dentin-bonding performance may be prudent.

### 3.3. Leachability of Unreacted Monomers from Copolymers and ACP Composites

**Figure 8:**
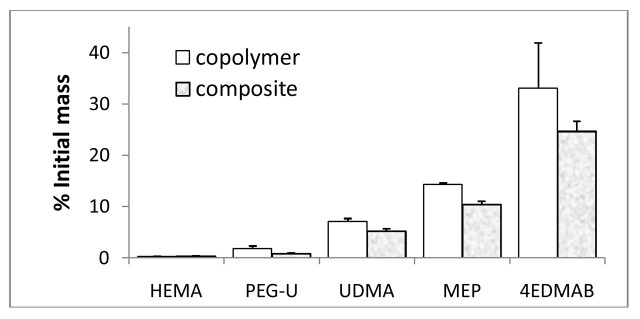
The levels of the unreacted monomers and photo-reductant detected by ^1^H NMR in acetone extracts of UDMA/PEG-U/HEMA/MEP copolymers and their g-ACP composites expressed as% of the initial amounts (mean value + SD). Number of repetitive measurements n = 3/group.

Typically, chromatographic techniques, such as high-performance liquid chromatography, gas chromatography and liquid chromatography-mass spectrometry are used to identify and quantify organic components that leach from dental composites. However, these techniques require longer sample preparation and results can be difficult to interpret. We have demonstrated that ^1^H NMR spectroscopy is a valuable technique that provides both qualitative and quantitative information on leachables without the burden of either sample preparation and/or data interpretation. The results of ^1^H NMR measurements performed with extracts collected from UDMA/PEG-U/HEMA/MEP (UPHM resin) copolymers and g-ACP/UPHM composites are shown in Figure 8.

The levels of the unreacted HEMA, MEP, PEG-U and UDMA detected in the copolymer and composite extracts ranged from 0.30% to 14.29% and 0.30% to 10.39% of the initial content, respectively. Photo-reductant, 4EDMAB, showed the highest leachability (33.07% and 24.66% in copolymer and composite extracts, respectively). However, when the composite leachability data are normalized with respect to the initial amount of the resins (100 mass % in copolymers *vs.* 60 mass % in the composites), differences between the copolymer and composite values become marginal indicating that introduction of ACP into UPHM resins does not affect the leachability of non-polymerized monomers and initiator components from the sealer. Furthermore, the DVC values attained in both UPHM copolymers and their g-ACP composites were generally very high (up to 90% [45]). In highly crosslinked UPHM polymers, the degree of mobility in polymer chains was relatively small and not many pathways existed for free monomer to leach out of the system. Therefore, both copolymer and composite systems are very likely above some DVC threshold over which mobility was very low and leachability has become practically constant.

The results of our NMR study were compared with published data on leachability of various commercial and experimental dental polymeric materials and reported in [83]. The reported concentrations for the same component varied significantly from study to study. This observation could possibly be explained by the fact that the compositional makeup of the resins as well as the type and the load of fillers differed greatly from material to material, and that a broad range of varying extraction conditions (type of solvent, duration of extraction, ratio of the specimen surface area/volume of the solvent) and identification methods were employed in the studies. Significantly, the levels of unreacted HEMA detected in our experimental materials ((0.03 to 0.04) mM) were much lower than the levels of HEMA released, for example, from resin composites (3.08 mM) or restorative resins ((0.16 to 0.38) mM). This finding is especially important having in mind the increased toxicity and adverse side effects reported for HEMA and TEGDMA monomers, which in the oral environment can be metabolized to methacrylic acid [84]. In addition, the level of the unreacted UDMA monomer extracted from UPHM copolymers or composites (0.49 mM) compares well with the concentrations of UDMA ((0.26 to 0.51) mM) reported for the wide range of the experimental UDMA/TEGDMA resins [85]. In both studies, no CQ was detected in the extracts of the polymerized specimens. In general, as a consequence of high DVC values attained in UPHM copolymers and their ACP composites, levels of the leachable components from the UPHM specimens do not exceed the levels of leachables seen in commercial materials. Our study also re-confirms that the elution of residual monomers from dental materials depends primarily on the composition of the resin, chemical characteristics of the leachable substances and the chemistry of the solvent. Because of the complex structure of the polymerized network, small molecular weight monomers such as HEMA, which because of its faster mobility would be expected to elute more than the higher weight molecules such as UDMA, do not necessarily do so.

### 3.4. In Vitro Cytotoxicity of Copolymers and ACP Composites

**Figure 9:**
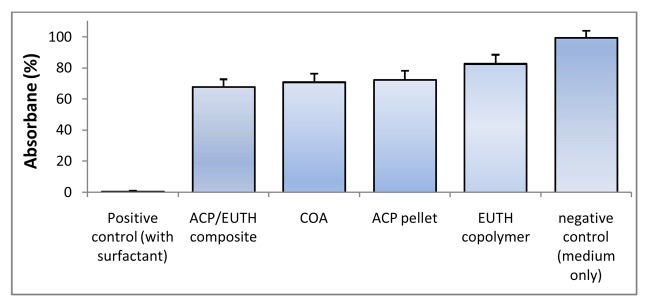
Dehydrogenase activity (Wst-1 assay; mean value + SD) for EBPADMA/ UDMA/TEGDMA/HEMA (EUTH) copolymer, m-ACP/EUTH composite and m-ACP powder compared to commercial orthodontic adhesive (COA) and positive control (0.1 mass % detergent).

Early interactions between the experimental copolymers and composites with osteoblastic cells were assessed in two *in vitro* studies. In the first study, undertaken as a part of the evaluation of the experimental LC m-ACP/EBPADMA/UDMA/TEGDMA/HEMA (EUTH resin) orthodontic cement, the cytotoxicity of EUTH copolymer, m-ACP/EUTH composite and m-ACP powder pellet specimens was evaluated by phase contrast microscopy and dehydrogenase activity assay. Cells cultured for 72 h in the extracts of all experimental materials (EUTH copolymer, ACP composite, ACP pellet, commercial orthodontic adhesive (COA; commercial control) and medium without any additives) exhibited a normal, spread, polygonal morphology (not shown here). In contrast, only the cell remnants were seen in a positive control, detergent-containing samples, indicating strong cytotoxicity of the detergent. Qualitatively, an approximately equivalent amount of cells was found in each experimental system suggesting no adverse cellular response to the tested specimens. However, quantitative tests of cell viability (Wst-1 assay; Figure 9) showed a mild to moderate drop in the extracts from the EUHT copolymer, m-ACP powder, m-ACP/EUHT and COA compared to the control medium. This reduction was attributed to leachable residual monomers and/or other unreacted species, and no attempt was made to correlate the cytotoxicity results with DVC values attained in these systems.

**Figure 10:**
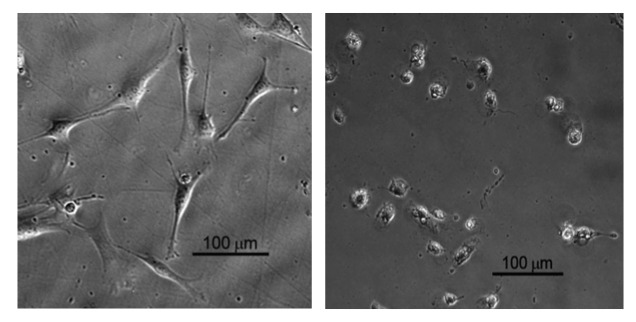
*In situ* cell morphology: spread and polygonal cells seen in the control medium and extracts of UPHM copolymer and g-ACP pellet (left); spherical cells seen in the extracts of g-ACP/UPHM and CES composites (right).

**Figure 11:**
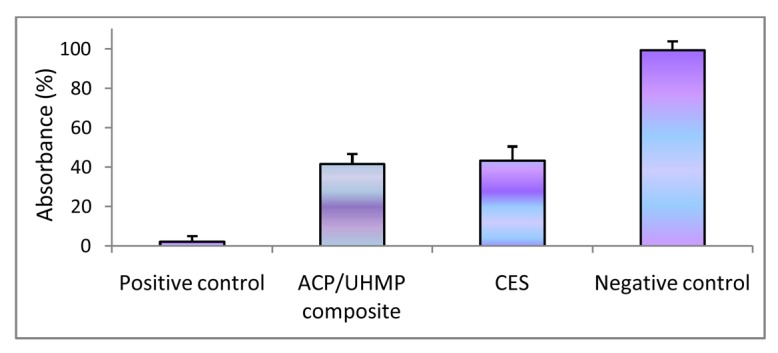
Dehydrogenase activity (MTT assay; mean value + SD) for ACP/UHMP composite, commercial endodontic sealer (CES), negative control (medium only) and positive control (medium + detergent).

In the second study, UDMA/PEG-U/HEMA/MEP (UPHM resin) copolymer, g-ACP pellets and the corresponding g-ACP/UPHM composites were extracted in media for 24 h, and murine pre-osteoblasts (MC3T3-E1) were then cultured in the extracts for 24 h. Extracts from a commercial endodontic sealer (CES) were used as a reference, and medium without any extract was used as a control. Second control was medium with added surfactant (1 mass %). The cell morphology was examined *in situ* at 24 h using optical microscopy. Cells in the extracts of UPHM copolymer, ACP pellet and the control medium without additive showed the spread, polygonal morphology (left image in Figure 10). However, the cells cultured in extracts of both g-ACP/UPHM and CES composite specimens exhibited a contracted, spherical morphology (right image in Figure 10). In addition to the morphological changes, cells exposed to the extracts from ACP/UHMP composites and CES also showed slow proliferation. Cells with the polygonal shape proliferated approximately 2.5 times faster than those contracted and sphere-shaped cells in 24 h according to cell viability tests using MTT methods **(**Figure 11). The copolymer and ACP powder did not induce the change individually because cells showed the polygonal morphology in their extracts. Furthermore, no significant differences were seen between DVC values attained in UPHM copolymer and its g-ACP composite counterparts, and the levels of unreacted monomers leaching from these materials (see Section 3.3). Further testing (possibly modified cell viability tests and cell proliferation experiments) will be required to better understand cellular responses to both the experimental ACP composite intended for endodontic application and the commercial control sealer. The spherical cell morphology and slow cell proliferation rate of MC3T3-E1 cell line has been reported in hydrogel scaffolds designed for bone regeneration, in which the preosteoblasts generated bone-like minerals [86]. It is also speculated that the morphology changes alternate the cytoskeletal tension on nucleus and nucleus organization and hence influence the mineralization of the osteoprogenitors [87].

The degree of cytotoxicity of various types of dental materials is routinely tested using a wide range of experimental protocols/designs (tested substances that stay in contact with cells for various time lengths are added to cell cultures as extracts of polymerized materials, as material specimens in direct contact with the cells, or on a dentin barrier or as individual components). The results reported in the literature are strongly model-dependent and the fact that they vary from little or no cytotoxicity to severe cytotoxicity with varying experimental procedures and exposure times accentuates the need for the improved standardized tests to obtain comparable results [88]. A comparison of the relative cytotoxicity of our experimental ACP/UPHM sealer with the cytotoxicity of various commercial dental resin composites, adhesives and root canal sealers [89] attest best to the above statement. Based on this comparison alone, regardless of the unwanted, and, at this point, unresolved effect on cell morphology, one could conclude that ACP/UPHM endodontic composite exhibits cytotoxicity comparable to a number of commercial products that have already found their use in dental clinics.

## 4. Conclusions

The comprehensive physicochemical evaluation of ACP composites is essential in order to understand the structure/composition/property relationships of these ACP materials and the complex mechanisms governing intra-composite ACP filler/polymer interactions. Our research seeks to stimulate studies that involve biocompatibility issues related to composites and promote the development of alternate polymeric chemistries and composite formulations that would yield materials based not only on suitable mechanical properties but also on improved biological performance. In broad terms, the biological risks of resin-based materials to the dentin-pulp complex originate from the toxicological properties of the materials themselves (direct bio-risks) and those arising from microbiological leakage (indirect bio-risks). Therefore, extensive leachability and cytotoxicity testing should be performed before the anti-demineralizing/remineralizing composites are tested in clinical trial with human subjects. It is expected that the findings of our continuing research will be useful as guideline(s) in future design of ACP/biodegradable polymeric materials for the generalized bone repair applications.

The spontaneous and uncontrolled agglomeration of particles during the ACP's synthesis typically yields a highly clustered ACP solid that disperses non-uniformly in matrix resins or their cured composites. The more favorable, narrower size distribution of ACP particles is routinely obtained through the mechanical treatment of ACP filler. ACP's grinding and, especially, milling rather than ACP's surface modification with various additives, typically results in better dispersion of these mechanically homogenized ACP fillers within polymer matrices and yield remineralizing ACP composites with improved mechanical stability.

Light-cure Bis-GMA-, EBPADMA- and UDMA-based copolymers and their ACP composites regularly achieve high degrees of vinyl conversion (DVC) when the hydrophilic HEMA is included as a co-monomer in the resin at relatively high content (HEMA ≥ 28 mass %). These higher DVC values are attributed to HEMA's high diffusivity and mono-functionality. As a consequence of high DVC, the experimental ACP composites undergo high shrinkage upon polymerization (PS). Their PS values typically exceed values reported for most commercial composite materials. This phenomenon can be attributed in part to the much lower filler level in our experimental materials (40 mass % ACP) compared to highly filled (up to 85 mass %) in conventional glass composites. A possible way to fabricate ACP composites with lower PS while maintaining satisfactory DVC and adequate mechanical properties would be the preparation of bulkier but relatively low viscosity experimental resins or inclusion of ring-opening monomers. In some formulations, such as UDMA/PEG-U/HEMA/MEP resins and their composites, relatively high PS is likely to be compensated by the significant post-polymerization hygroscopic expansion (HE) of these materials upon immersion in aqueous medium. Beneficial aspects of HE have indeed been demonstrated by other researchers for different composite materials.

In the case of ACP composite materials, besides the water-polymer interactions, strong water-ACP filler interactions additionally contribute to the overall water sorption (WS) profiles. Besides affecting the mechanical integrity of the composites, water diffusion affects the kinetics of the intra-composite ACP conversion to HAP and ultimately determines the remineralizing capacity of these materials. Generally, higher remineralizing potential is attained when the polymer phase contains EBPADMA or UDMA as base monomers and HEMA or HEMA plus PEG-U as co-monomers. The most probable reason for higher ion releases obtained with these base monomers is a more open cross-linked network structure of their resin matrix. Hydrophilic HEMA-enriched matrices generally increase internal mineral saturation by allowing the uptake of more water and/or better accessibility of ACP filler to the water already entrained.

In addition to material factors (filler type and/or content, resin type and composition, polymerization mode), processing factors such as cavity configuration (C-factor) need to be considered when assessing polymerization shrinkage stress (PSS) development in composites. However, the apparent discrepancies between the PS and PSS in relation to the composition of the resin matrix have yet to be resolved.

The ^1^H NMR is a valuable technique that provides both qualitative and quantitative information on leachables without the burden of elaborate sample preparation and/or data interpretation. Leachability of the unrectaed monomers from the experimental ACP sealer is apparently controlled by the highly cross-linked UDMA/PEG-U/HEMA/MEP resin network and unaffected by the incorporation of bioactive ACP into the resin. The maximum levels of leachables from our experimental composite are within or below the concentration ranges reported for the commercial counterparts. Additional cellular research appears necessary in order to assess possible biological effects of the components released from UPHM-based materials.

The chemical structure/property relationships of the monomers, compositional differences involving polymers and polymerization initiation systems, and the attainable DVC values are important factors that determine the cytotoxicity of the polymeric ACP composites. *In vitro* cytotoxicity tests comparing the experimental material with the representative commercial control are a good predictor of the new material's suitability for the intended applications and should be one of the main parameters when considering its recommendation for clinical trials.

The results of the above discussed studies are useful guidelines in designing remineralizing ACP composites for different dental utilities. The lessons learned from these studies may be essential in the future development of amorphous calcium phosphate-based biomaterials intended for general bone repair.

## **Appendix 1.** List of acronyms used throughout the manuscript

### Abbreviations

ACP: amorphous calcium phosphate
ADAF: American Dental Association Foundation
AES: atomic emission spectroscopy
Ag-ACP: silver-modified ACP
Al-ACP: aluminum-modified ACP
ALP: alkaline phosphatase
am-ACP: as made ACP
ANOVA: analysis of variance
APTMS: 3-aminopropyltrimethoxysilane
APTMS-ACP: APTMS silanized ACP
ASTM: American Society for Testing and Materials
BFS: biaxial flexural strength
BHT: butylated hydroxyl toluene
Bis-GMA: 2,2-bis[p-(2-hydroxy-3-methacryloxypropoxy)phenyl]propane
BPO: benzyl peroxide
BTHZ: Bis-GMA/TEGDMA/HEMA/ZrDMA resin
CC: chemical cure
C factor: cavity configuration factor
CaP: calcium phosphate
CES: commercial endodontic sealer
COA: commercial orthodontic adhesive
CQ: camphorquinone
4265 Darocur: commercial polymerization initiating system
DC: dual cure
DCPD: dicalcium phosphate dihydrate
DHEPT: 2,2′-dihydroxyethyl-p-toluidine
d_m_: median particle diameter
DMSO: dimethylsulfoxide
DVC: degree of vinyl conversion
EBPADMA: ethoxylated bisphenol A dimethacrylate
4EDMAB: ethyl-4-N,N-dimethylamino benzoate
EDTA: ethylenediamine tetraacetic acid
ETHM: EBPADMA/TEGDMA/HEMA/MEP resin
EUTH: EBPADMA/UDMA/TEGDMA/HEMA resin
FAP: fluoroapatite
Fe^2+^-ACP: iron (II)-modified ACP
Fe^3+^-ACP: iron (III)-modified ACP
FTIR: Fourier transform infrared spectroscopy
FTIR-m: FTIR micro-spectroscopy
ΔG^o^: Gibbs free energy
g-ACP: ground ACP
HAP: hydroxyapatite
HE: hygroscopic expansion
HEMA: 2-hydroxyethyl methacrylate
HEPES: 4-(2-hydroxyethyl)-1-piperazineethane sulfonic acid
HmDMA: hexamethylene dimethacrylate
IAP: ion activity product
1850 Irgacure: commercial polymerization initiating system
369 Irgacure: commercial polymerization initiating system
K_sp_: thermodynamic solubility product
LC: light cure
m-ACP: milled ACP
MEP: methacryloyloxyethyl phthalate
MPTMS: methacryloxypropyltrimethoxysilane
MPTMS-ACP: MPTMS silanized ACP
MTT: dehydrogenase activity assay
n: number of specimens (or repetitive experiments)
NIDCR: National Institute of Dental and Craniofacial Research
NIR: near infrared spectroscopy
NIST: National Institute of Standards and Technology
NMR: nuclear magnetic resonance
OCP: octacalcium phosphate pentahydrate
PBS: phosphate buffered saline solution
PEG-U: poly(ethylene glycol) extended urethane dimethacrylate
PEO: poly(ethylene oxide)
PEO-ACP: PEO modified ACP
PRC: Paffenbarger Research Center
PS: polymerization shrinkage
PSD: particle size distribution
PSS: polymerization shrinkage stress
R: ideal gas constant
RH: relative humidity
SBS: shear bond strength
SEM: scanning electron microscopy
SD: standard deviation
Si-ACP: silicon-modified ACP
T: absolute temperature
TCP: tricalcium phosphate
TEGDMA: triethylene glycol dimethacrylate
TGA: thermogravimetric analysis
TRITON: alkyl aryl polyether alcohol (nonionic surfactant)
TWEEN: poly(oxyethylene) sorbitan monolaureate (nonionic surfactant)
UPHM: UDMA/PEG-U/HEMA/MEP resin
WS: water sorption
Wst1: mitochondrial dehydrogenase activity assay
XRD: X-ray diffraction
Zn-ACP: zinc-modified ACP
ZONYL FSN: non-ionic fluoro-surfactant
ZONYL FSP: anionic fluoro-surfactant
Zr-ACP: zirconia-modified ACP
ZrDMA: zirconyl dimethacrylate

## References

[1] Dorozhkin S.V. (2010). Amorphous calcium (ortho)phosphates. Acta Biomater..

[2] Dorozhkin S.V. (2010). Calcium orthophosphates as bioceramics: State of the art. J. Funct. Biomater..

[3] Wang L., Nancollas G.H. (2009). Pathways to biomineralization and biodemineralization of calcium phosphates: The thermodynamic and kinetic controls. Dalton Trans..

[4] Putlyaev V.I., Safronova T.V. (2006). A new generation of calcium phosphate biomaterials: The role of phase and chemical compositions. Glass Ceram..

[5] Vallet-Regi M., Gonzales-Calbert J.M. (2004). Calcium phosphates as substitution of bone tissues. Prog. Solid State Chem..

[6] Hench L.L., Xynos I.D., Polak J.M. (2004). Bioactive glasses for *in situ* tissue regeneration. J. Biomater. Sci. Ploym. Ed..

[7] Barrere F., van Blitterswijk C.A., de Groot K. (2006). Bone regeneration: Molecular and cellular interactions with calcium phosphate ceramics. Int. J. Nanomed..

[8] Kartsogiannis V., Ng K.W. (2004). Cell lines and primary cell cultures in the study of bone cell biology. Mol. Cell. Endocrinol..

[9] Annaz B., Hing K.A., Kayser M., Buckland T., di Silvio L. (2004). An ultrastructural study of cellular response to variation in porosity in phase-pure hydroxyapatite. J. Microsc..

[10] Devlin H., Sloan P. (2002). Early bone healing events in the human extraction socket. Int. J. Oral Maxillofac. Surg..

[11] Radin S., Ducheyne P., Berthold P., Decker S. (1998). Effect of serum proteins and osteoblasts on the surface transformation of a calcium phosphate coating: A physicochemical and ultrastructural study. J. Biolmed. Mater. Res..

[12] De Bruijn J.D., Flach J.S., Leenders H., van den Brink J., van Blitterswijk C.A. (1992). Degradation and interface characteristics of plasma-sprayed hydroxyapatite coatings with different crystallinities. Bioceramics.

[13] Midy V., Dard M., Hollande E. (2001). Evaluation of the effect of three calcium phosphate powders on osteoblast cells. J. Mater. Sci. Mater. Med..

[14] Siebers M.C., Walboomers X.F., Leeuwenburgh S.C.G., Wolke J.C.G., Jansen J.A. (2004). Electrostatic spray deposition (ESD) of calcium phosphate coatings, an *in vitro* study with osteoblast-like cells. Biomaterials.

[15] Wang C., Duan Y., Markovic B., Barbara J., Howlett C.R., Zhang X., Zreiqat H. (2004). Phenotypic expression of bone-related genes in osteoblasts grown on calcium phosphate ceramics with different phase compositions. Biomaterials.

[16] Arinzeh T.L., Tran T., Mcalary J., Daculsi G. (2005). A comparative study of biphasic calcium phosphate ceramics for human mesenchymal stem-cell induced bone formation. Biomaterials.

[17] Adams C.S., Mansfield K., Perlot R.L., Shapiro I.M. (2001). Matrix regulation of skeletal cell apoptosis—Role of calcium and phosphate ions. J. Biol. Chem..

[18] Dvorak M.M., Siddiqua A., Ward D.T., Carter H., Dallas S.H., Nemeth E.F., Riccardi D. (2004). Physiological changes in extracellular calcium concentration directly control osteoblast function in the absence of claciotropic hormones. Proc. Natl. Acad. Sci. USA.

[19] Redey S.A., Nardin M., Bernache-Assolant D., Delannoy L.S., Marie P.J. (2000). Behavior of human osteoblastic cells on stoichiometric hydroxyapatite and type A carbonate apatite: Role of surface energy. J. Biolmed. Mater. Res..

[20] Lu X., Leng Y. (2003). Quantitative analysis of osteoblast behavior on microgrooved hydroxyapatite and titanium substrata. J. Biolmed. Mater. Res..

[21] Chou Y.F., Dunn J.C.Y., Wu B.M. (2005). *In vitro* response of MC3T3-E1 preosteoblast within three-dimensional apatite-coated PLGA scaffolds. J. Biolmed. Mater. Res..

[22] De Bruijn J.D., Bovell Y.P., Davies J.E., van Blitterswijk C.A. (1994). Osteoclastic resorption of calcium phosphates is potentiated in postosteogenic culture conditions. J. Biolmed. Mater. Res..

[23] Chou Y.F., Huang W., Dunn J.C.Y., Miller T.A., Wu B.M. (2005). The effect of biomimetic apatite structure on osteoblast viability, proliferation and gene expression. Biomaterials.

[24] Heyman D., Pradal G., Benahmed M. (1999). Cellular mechanisms of calcium phosphate ceramics degradation. Histol. Histopathol..

[25] Lu J., Descamps M., Dejon J., Koubi G., Hardonin P., Lemaitre J., Proust J.P. (2002). The biodegradation mechanism of calcium phosphate biomaterials in bone. J. Biolmed. Mater. Res..

[26] Zerbo I.R., Bronckers A.L.J.J., de Lange G. (2005). Localization of osteogenic and osteoclastic cells in porous [beta]-tricalcium phosphate particles used for human maxillary sinus floor elevation. Biomaterials.

[27] Haders D.J., Kazanecki C.C., Denhardt D.T., Riman R.E. (2010). Crystallographically engineered, hydrothermally crystallized hydroxyapatite films: An *in vitro* study of bioactivity. J. Mater. Sci. Mater. Med..

[28] Reynolds E.C., Black C.L., Cai F., Cross K.J., Eakins D., Huq N.L., Morgan M.V., Nowicki A., Perich J.W., Riley P.F. (1999). Advances in enamel remineralization: Casein phosphopeptide-amorphous calcium phosphate. J. Clin. Dent..

[29] Ten Cate J.M. (1999). Current concepts on the theories of the mechanism of action of fluoride. Acta Odontol. Scand..

[30] Kashet S. (1999). Historical review of remineralization research. J. Clin. Dent..

[31] Featherstone J.D. (1994). Fluoride, remineralization and root caries. Am. J. Dent..

[32] Chow L.C., Vogel G.L. (2001). Enhancing remineralization. Oper. Dent..

[33] Reynolds E.C., Cai F., Shen P., Walker G.D. (2003). Retention in plaque and remineralization of enamel lesions by various forms of calcium in a mouthrinse or sugar-free chewing gum. J. Dental Res..

[34] Reynolds E.C., Cai F., Cohrane N.J., Shen P., Walker G.D., Morgan M.V., Reynolds C. (2008). Fluoride and casein phosphopeptide-amorphous calcium phosphate. J. Dental Res..

[35] Skrtic D., Antonucci J.M., Eanes E.D. (2003). Amorphous calcium phosphate-based bioactive polymeric composites for mineralized tissue regeneration. J. Res. Natl. Inst. Stand. Technol..

[36] Skrtic D., Antonucci J.M., Lechov M., Prandzheva S. (2010). Design, characterization and evaluation of biomimetic polymeric dental composites with remineralization potential. Encyclopedia of Polymer Composites: Properties, Performance and Applications.

[37] Langhorst S.E., O'Donnell J.N.R., Skrtic D. (2009). *In vitro* remineralization effectiveness of polymeric ACP composites: Quantitative micro-radiographic study. Dental Mater..

[38] Tung M.S., Eichmiller F.C. (1999). Dental applications of amorphous calcium phosphates. J. Clin. Dent..

[39] Santerre J.P., Shajii L., Leung B.W. (2001). Relation of dental composite formulations to their degradation and the release of hydrolyzed polymeric-resin-derived products. Crit. Rev. Oral Biol. Med..

[40] Pelka M., Distle R.W., Petshelt A. (1999). Elution parameters and HPLC-detection of single components from resin composite. Clin. Oral Investig..

[41] Spahl W., Budzikiewicz H., Geurtsen W. (1998). Determination of leachable components from four commercial dental composites by gas and liquid chromatography/mass spectrometry. J. Dent..

[42] Antonucci J.M., Skrtic D. (2006). Physicochemical and mechanical evaluation of cation-modified ACP acrylic resin composites. Polym. Prepr..

[43] Antonucci J.M., Liu D.W., Skrtic D. (2007). Amorphous calcium phosphate based composites: Effect of surfactants and poly(ethylene) oxide on filler and composite properties. J. Dispers. Sci. Technol..

[44] Antonucci J.M., Skrtic D., Ramalingam M., Tiwari A. (2011). Bioactive and biocompatible polymeric composites based on amorphous calcium phosphate. Integrated Biomaterials for Medical Applications, Vol. 1: Biomaterials—Protocols and Techniques.

[45] O'Donnell J.N.R., Skrtic D. (2009). Degree of vinyl conversion, polymerization shrinkage and stress development in experimental endodontic composites. J. Biomim. Biomater. Tissue Eng..

[46] Lee S.Y., Regnault W.F., Antonucci J.M., Skrtic D. (2007). Effect of particle size of an amorphous calcium phosphate filler on the mechanical strength and ion release of polymeric composites. J. Biomed. Mater. Res..

[47] Stansbury J.W., Dickens S.H. (2001). Network formation and compositional drift during photo-initiated copolymerization of dimethacrylate monomers. Polymer.

[48] (1996). ASTM Standard F394-78: Standard Test Method for Biaxial Strength (Modulus of Rupture) of Ceramic Substrates.

[49] Skrtic D., Antonucci J.M., Eanes E.D., Eidelman N. (2004). Dental composites based on hybrid and surface-modified amorphous calcium phosphates—A FTIR microspectroscopic study. Biomaterials.

[50] Lu H., Stansbury J.W., Dickens S.H., Eichmiller F.C., Bowman C.N. (2004). Probing the origins and control of shrinkage stress in dental resin-composites: I. Shrinkage stress characterization technique. J. Mater. Sci. Mater. Med..

[51] Schumacher G.E., Antonucci J.M., O'Donnell J.N.R., Skrtic D. (2007). The use of amorphous calcium phosphate composites as bioactive materials and their effect on the strength of the composite/adhesive/dentin bond. J. Am. Dental Assoc..

[52] O'Donnell J.N.R., Schumacher G.E., Antonucci J.M., Skrtic D. (2009). Adhesion of amorphous calcium phosphate composites bonded to dentin: A study in failure modality. J. Biolmed. Mater. Res..

[53] Simon C.G., Antonucci J.M., Liu D.W., Skrtic D. (2005). *In vitro* cytotoxicity of amorphous calcium phosphate composites. J. Bioact. Compat. Polym..

[54] Ishiyama M., Shiga M., Sasamoto K., Mizoguchi H., He P.G. (1993). A new sulfonated tetrazolium salt that produces a highly water-soluble formazan dye. Chem. Pharm. Bull..

[55] Amjad Z. (2004). Inhibition of the amorphous calcium phosphate phase transformation reaction by polymeric and non-polymeric inhibitors. Phosphorus Res. Bull..

[56] Ofir P.B.Y., Govrin-Lipman R., Garti N., Furedi-Milhofer H. (2004). The influence of polyelectrolytes on the formation and phase transformation of amorphous calcium phosphate. Cryst. Growth Des..

[57] Eanes E.D., Amjad Z. (1998). Amorphous calcium phosphate: Thermodynamic and kinetic considerations. Calcium Phosphates in Biological and Industrial Systems.

[58] Skrtic D., Antonucci J.M., Eanes E.D., Brunworth R.T. (2002). Silica- and zirconia-hybridized amorphous calcium phosphate. Effect on transformation to hydroxyapatite. J. Biolmed. Mater. Res..

[59] Venhoven B.A.M., de Gee A.J., Davidson C.L. (1993). Polymerization contraction and conversion of light-curing Bis-GMA based methacrylate resins. Biomaterials.

[60] Labella R., Lambrecths P., van Meerbeck B., Vanherle G. (1999). Polymerization shrinkage and elasticity of flowable composites and filled adhesives. Dental Mater..

[61] Guggenbarger R., Weinmann W. (2000). Exploring beyond methacrylates. Am. J. Dent..

[62] Tilbrook D.A., Clarke R.L., Howle N.E., Braden M. (2000). Photocurable epoxy-polyol matrices for use in dental composites. Biomaterials.

[63] Arima T., Hamada T., Mccabe J.F. (1995). The effects of cross-linking agents on some properties of HEMA-based resins. J. Dental Res..

[64] Garcia-Fiero J.L., Aleman J.V. (1982). Sorption of water by epoxide prepolymers. Macromolecules.

[65] Skrtic D., Antonucci J.M. (2007). Dental composites based on amorphous calcium phosphate—Resin composition/physicochemical properties study. J. Biomater. Appl..

[66] Antonucci J.M., Regnault W.F., Skrtic D. (2010). Polymerization shrinkage and polymerization stress development in amorphous calcium phosphate/urethane dimethacrylate polymeric composites. J. Compos. Mater..

[67] Momoi Y., McCabe J.F. (1994). Hygroscopic expansion of resin based composites during 6 months water storage. Br. Dental J..

[68] Huang C., Tay F.R., Cheung G.S.P., Kei L.H., Wei S.H.Y., Pashley D.H. (2002). Hygroscopic expansion of a compomer and a composite on artificial gap reduction. J. Dent..

[69] Ferracane J.L. (2005). Developing a more complete understanding of stresses produced in dental composites during polymerization. Dental Mater..

[70] Braga R.R., Ferracane J.L. (2002). Contraction stress related to degree of conversion and reaction kinetics. J. Dental Res..

[71] Calheiros F.C., Braga R.R., Kawano Y., Ballester R.Y. (2004). Relationship between contraction stress and degree of conversion in restorative composites. Dental Mater..

[72] Stansbury J.W., Trujillo-Lemon M., Lu H., Ding X., Lin Y., Ge J. (2005). Conversion-dependent shrinkage stress and strain in dental resins and composites. Dental Mater..

[73] Kleverlaan C.J., Feilzer A.J. (2005). Polymerization shrinkage and contraction stress of dental resin composites. Dental Mater..

[74] Choi K.K., Ruy G.J., Choi S.M., Lee M.J., Park S.J., Ferracane J.L. (2004). Effects of cavity configuration on composite restoration. Oper. Dent..

[75] Uno S., Tanaka T., Inoue S., Sano S. (1999). The influence of configuration factors on cavity adaptation in compomer restorations. Dental Mater..

[76] Feilzer A.J., de Gee A.J., Davidson C.L. (1990). Quantitative determination of stress reduction by flow in composite restorations. Dental Mater..

[77] Alster D., Feilzer A.J., de Gee A.J., Davidson C.L. (1997). Polymerization contraction stress in thin resin composite layers as a function of layer thickness. Dental Mater..

[78] Choi K.K., Condon J.R., Ferracane J.L. (2000). The effects of adhesive thickness on polymerization contraction stress of composite. J. Dental Res..

[79] Watts D.C., Satterthwaite J.D. (2008). Axial shrinkage stress depends upon both c-factor and composite mass. J. Dental Res..

[80] Munksgaard E.C., Hansen E.K., Kato H. (1987). Wall-to-wall polymerization contraction of composite resins *versus* filler content. Scand. J. Dent..

[81] Antonucci J.M., Giuseppetti A.A., O'Donnell J.N.R., Schumacher G.E., Skrtic D. (2009). Polymerization stress development in dental composites: Effect of cavity design factor. Materials.

[82] Antonucci J.M., O'Donnell J.N.R., Schumacher G.E., Skrtic D. (2009). Amorphous calcium phosphate composites and their effect on the composite/adhesive/dentin bond. J. Adhes. Sci. Technol..

[83] Davis C.H., O'Donnell J.N.R., Skrtic D. (2011). Determination of leachable components from an experimental ACP endodontic sealer by ^1^H NMR. Dental Mater..

[84] Durner J., Walther U.I., Zaspel J., Hickel R., Reichl F.X. (2010). Metabolism of TEGDMA and HEMA in human cells. Biomaterials.

[85] Floyd C.J.E., Dickens S.H. (2006). Network structure of Bis-GMA- and UDMA-based resin systems. Dental Mater..

[86] Chatterjee K., Lin-Gibson S., Wallace W.E., Parekh S.H., Lee Y.J., Cicerone M.T., Young M.F., Simon C.G. (2010). The effect of 3D hydrogel scaffold modulus on osteoblast differentiation and mineralization revealed by combinatorial screening. Biomaterials.

[87] Dalby M.J., Gadegaard N., Tare R., Andar A., Riehle M.O., Herzyk P., Wilkinson C.D.W., Oreffo R.O.C. (2007). The control of human mesenchymal cell differentiation using nanoscale symmetry and disorder. Nat. Mater..

[88] Franz A., Kőnig F., Lucas T., Watts D.C., Schedle A. (2009). Cytotoxic effects of dental bonding substances as a function of degree of conversion. Dental Mater..

[89] O'Donnell J., Sun J., Skrtic D. (2011). *In vitro* cytotoxicity of the experimental ACP endodontic sealer. Int. Assn. Dent. Res..

